# Dose-dependent pharmacokinetics and acute effects of intravenous bolus *N,N-*dimethyltryptamine: double-blind, randomized versus open-label dose-escalation administration study in healthy participants

**DOI:** 10.1038/s41398-026-03987-7

**Published:** 2026-03-27

**Authors:** Livio Erne, Lorenz Mueller, Isabelle Straumann, Bislim Ademaj, Anne Eckert, Ina Vukalovic, Jan Valenta, Dino Luethi, Matthias E. Liechti, Severin B. Vogt

**Affiliations:** 1 Clinical Pharmacology and Toxicology, Department of Biomedicine and Department of Clinical Research, University Hospital Basel, Basel, Switzerland; 2 Department of Pharmaceutical Sciences, University of Basel, Basel, Switzerland; 3 Psychiatric University Hospital, University of Basel, Basel, Switzerland; 4 Transfaculty Research Platform Molecular and Cognitive Neuroscience, University of Basel, Basel, Switzerland

**Keywords:** Psychology, Neuroscience

## Abstract

*N*,*N*-dimethyltryptamine (DMT) is a serotonergic psychedelic that produces short-lived peak effects when administered intravenously as a bolus dose. Initial trials suggested therapeutic effects of DMT in depressive disorders. However, systematic data on dose-dependent pharmacokinetics and acute effects of intravenous bolus DMT administration are currently lacking. We used a double-blind, randomized, placebo-controlled, crossover design in 20 healthy participants who received placebo and DMT (5, 10, 15, and 20 mg) within a single test session. In a separate study arm, we used an open-label, DMT dose-escalation administration design where 16 participants received stepwise increases of 5 mg DMT until reaching a maximally tolerated dose (max. 25 mg). Outcome measures included subjective effects, autonomic effects, adverse effects, and pharmacokinetics that were assessed up to 55 min after each bolus administration. Bolus DMT doses induced very strong subjective effects with a very rapid onset and peak within the first 2 min after administration. Subjective effects declined quickly and subsided completely within 12–30 min, consistent with the short elimination half-life of approximately 6–7 min. A ceiling effect for peak subjective effects was reached at the 15 mg dose. No tolerance was observed to acute effects of DMT. The tolerability markedly improved with open-label dose-escalation compared with double-blinded, randomized administration, and at equivalent dose levels, subjective effects were rated as less intense. These findings highlight the impact of blinding and expectancy on the subjective experience and suggest that individual dose-escalation may improve tolerability and help with dose selection for future DMT studies.

## Introduction

*N*,*N*-dimethyltryptamine (DMT) is a serotonergic psychedelic that is known for its intense and short-lasting psychoactive effects. When taken orally, DMT is degraded by monoamine oxidase (MAO), rendering it inactive unless it is co-administered with an MAO inhibitor, such as in the traditional Amazonian brew ayahuasca [1, 2]. To circumvent first-pass metabolism and avoid longer lasting effects when combined with MAO inhibition, modern clinical research favors parenteral routes of DMT administration, such as intravenous [3–9] or inhalation [10].

The first controlled intravenous bolus DMT administration studies were conducted in the 1990s by Strassman, who administered bolus doses from 0.05–0.4 mg/kg over 45 s in participants with extensive hallucinogen experience [11–13]. More recent research used continuous infusions of DMT or an initial loading bolus dose followed by infusions [3–6, 9, 14]. One study reported lower tolerability of intravenous bolus doses of 15 and 25 mg compared with continuous infusions of 0.6 and 1.0 mg/min, attributing the difference primarily to an intense and rapid onset of effects that was associated with bolus administration [3]. However, two recent small open-label clinical trials reported rapid and promising antidepressant effects of DMT when it was administered as intravenous bolus doses in patients with major depressive disorder [8] or vaporized DMT in patients with treatment-resistant depression [15]. Similarly, intranasal and inhaled 5-methoxy-DMT, a close analog of DMT, are being evaluated [16, 17]. These investigations suggest that administration methods that produce rapid and intense peak effects may hold therapeutic potential. However, the pharmacokinetics, acute effects, and safety- of intravenous DMT bolus administration across different doses have not been systematically investigated in a controlled, within-subjects design in participants with limited or no history of psychedelic use. To fill this gap, the present study characterized dose-dependent acute subjective, autonomic, and adverse effects and pharmacokinetics of bolus DMT administration using a randomized, placebo-controlled, within-subjects design. Additionally, we included a separate open-label, dose-escalation arm to assess how different study designs and associated blinding and expectancies influence subjective responses. The open-label dose-escalation arm also evaluated the feasibility of gradually increasing the DMT dose within the same day to a maximally tolerated level, an approach that might be applied in future clinical and therapeutic settings.

## Methods and materials

### Study design

The study comprised two independent study arms: a blinded, randomized arm and an open-label, dose-escalation arm. After successful screening, participants were allowed to select their preferred study arm. The randomized arm used a double-blind, placebo-controlled, crossover design to investigate responses to (*i*) placebo, (*ii*) 5 mg DMT, (*iii*) 10 mg DMT, (*iv*) 15 mg DMT, and (*v*) 20 mg DMT. Participants were informed that they would receive five bolus doses in randomized order, consisting of placebo (saline) and DMT doses of 5, 10, 15, and 20 mg, and that higher doses could produce rapid onset, very intense, but short-lived subjective effects. The dose-escalation arm used an open-label design, in which the participants first received placebo, followed by ascending doses of DMT, starting at 5 mg and increasing in 5 mg increments up to a maximum of 25 mg. After each dose, the participants could decide whether to stop or proceed to the next dose level. The dose selection was based on prior empirical data and pharmacodynamic considerations. In a previous clinical trial, an intravenous DMT dose of 25 mg produced very strong subjective effects with limited tolerability [3]. Moreover, a ceiling effect was present between the 15 mg and 25 mg dose, reflected by a less-than-proportional increase in subjective intensity between these two doses. Accordingly, a maximum target dose of 20 mg was selected for the randomized arm, while doses up to 25 mg were permitted in the dose-escalation arm to account for interindividual differences (e.g., participants experiencing lower subjective effects or demonstrating higher tolerability). The washout periods between dose administrations were 1 h. The washout period was based on pharmacokinetic considerations and prior empirical evidence. Given DMT’s short elimination half-life of approximately 5–7 min, most of the drug was expected to be eliminated within 1 h. In addition, previous clinical studies have demonstrated that subjective effects fully subside within approximately 20–30 min following the cessation of a continuous intravenous DMT infusion, even at very high doses [3, 4]. In cases of an intense or negative prior bolus experience, the participants were allowed to extend the time between two bolus administrations by a maximum of 30 min. All experimental protocols were approved by the Ethics Committee of Northwest and Central Switzerland (BASEC 2022-01224) and the Swiss Federal Office for Public Health. The study was conducted in accordance with the Declaration of Helsinki and International Conference on Harmonization Guidelines in Good Clinical Practice. Written informed consent was obtained from all participants prior to participation. All methods were performed in accordance with the relevant guidelines and regulations. The study was registered at ClinicalTrials.gov (NCT05695495).

### Participants

Thirty-six healthy participants were recruited by word of mouth or from a pool of volunteers who had contacted our research group because they were interested in participating in a clinical trial that investigated psychedelics. Of these, 20 participants (10 men and 10 women; mean age ± SD: 34 ± 9 years; range: 25–56 years) and 16 participants (8 men and 8 women; mean age ± SD: 39 ± 13 years; range: 25–65 years) were included in the randomized arm and dose-escalation arm, respectively. The participants were allowed to choose their preferred study arm. All participants provided written informed consent and were paid for their participation. Exclusion criteria were age < 25 years or > 65 years, pregnancy (urine pregnancy test at screening and before the test session), personal or family (first-degree relative) history of major psychiatric disorders (assessed by a semi-structured clinical interview based on the *Diagnostic and Statistical Manual of Mental Disorders*, 5th edition [18], by a trained physician), the use of medications that may interfere with the study medication (e.g., antidepressants, antipsychotics, or sedatives), chronic or acute physical illness (e.g., abnormal physical exam, electrocardiogram, or hematological and chemical blood analyses), tobacco smoking (> 10 cigarettes/day), lifetime prevalence of psychedelic drug use > 20 times, illicit drug use within the last 2 months (except for Δ^9^-tetrahydrocannabinol), and illicit drug use during the study period (screened by urine drug tests). The participants were asked to consume no more than 20 standard alcoholic drinks/week and have no more than one drink on the day before the test sessions. Four participants (20%) in the randomized arm and six participants (37%) in the dose-escalation arm were psychedelically naive. Detailed data on lifetime substance use are provided in the Supplement (Supplementary Table S1).

### Study drugs

DMT hemifumarate (99.9% high-performance liquid chromatography purity, ReseaChem GmbH, Burgdorf, Switzerland) was formulated according to Good Manufacturing Practice and prepared in sterile vials that contained 5 mg/ml DMT in 1 ml of purified water. The exact analytically confirmed DMT hemifumarate content of the vials (mean ± SD) was 4.76 ± 0.05 mg (*n* = 10 samples). The stability of DMT in the vials was confirmed for the study duration. Placebo consisted of identical vials that were filled with sterile water only. In the randomized study arm, each bolus consisted of the content of four vials that contained either 5 mg/ml DMT or placebo, which were aspirated into a syringe and diluted with saline (0.9% NaCl) to a volume of 20 ml. In the dose-escalation study arm, one to five open-label DMT vials of 5 mg/ml or one placebo vial, depending on the target dose, were aspirated into a syringe and diluted with saline (0.9% NaCl) to a volume of 20 ml. The 20 ml intravenous bolus dose was administered over 45 s. After each bolus administration and at the end-of-study visit, the participants in the randomized study arm were asked to retrospectively guess their treatment assignment to evaluate blinding.

### Study procedures

The study included a screening visit, one 6-h test session, and an end-of-study visit. The sessions were conducted in a calm hospital room. One research participant and two investigators were present during each session. The test sessions began at approximately 9:00 AM. A urine sample was taken to verify abstinence from drugs of abuse, and a urine pregnancy test was performed in women before each session. The participants then underwent baseline measurements. The first DMT or placebo dose was administered at 10:00 AM, followed by the next administration 1 h later. The outcome measures were repeatedly assessed for 55 min after each bolus administration. The participants were sent home approximately 15 min after the last measurement.

### Subjective drug effects

Subjective effects were assessed repeatedly using subjective effect scales 1 h before and 0, 2, 4, 8, 12, 18, 30, and 55 min after the administration of a bolus dose. Subjective effect scales included verbal ratings of “any drug effect,” “good drug effect,” “bad drug effect,” and “fear” (Likert scale; 0–10 for no to maximal effect). The short version of the Altered States of Consciousness scale (3D-ASC), comprising 42 core items from the 5D-ASC scale [19, 20], was administered after each bolus dose to retrospectively assess psychedelic peak effects once all subjective effects had subsided. Psychedelic experiences were also similarly assessed after each bolus dose using the 48-item Psychedelic Experience Scale (PES48), which includes the widely used Mystical Experience Questionnaire [21]. Additionally, near-death experience-like effects were assessed retrospectively using the Near-Death Experience Content (NDE-C) scale after each bolus dose [22]. Subjective effect measurements are described in more detail in the Supplementary Methods online.

### Autonomic and adverse effects

Blood pressure and heart rate were measured at baseline and 2, 4, 8, 12, 18, 30, and 55 min after bolus administration. Acute adverse effects were assessed 1 h before administration of the first bolus dose and at the end of the session day 30 min after administration of the last bolus dose for all doses together using the List of Complaints [23].

### Plasma DMT concentrations

Blood was collected into lithium heparin tubes at baseline and 2, 4, 8, 12, 18, 30, and 55 min after bolus administration. The blood samples were immediately centrifuged, and plasma was subsequently stored at −20 °C and later at −80 °C until analysis. Plasma concentrations of DMT and its metabolites were determined by high-performance liquid chromatography-tandem mass spectrometry using a validated method as previously described [24].

### Pharmacokinetic analyses

Pharmacokinetic parameters were estimated using non-compartmental methods as described previously [3, 25]. Analyses were conducted using Phoenix WinNonlin 8.4 (Certara, Princeton, NJ, USA).

### Data analysis

Peak (E_max_) or peak change from baseline (ΔE_max_) values were determined for repeated measures. For the randomized study arm, the values were then analyzed using analysis of variance, with dose as the within-subjects factor, followed by the Tukey post hoc test. The criterion for significance was *p* < 0.05. For the comparison of outcomes between the randomized and dose-escalation arms, the Wilcoxon Rank Sum test was used; however, these p-values should be considered exploratory, as the study was powered for within-subject rather than between-subject comparisons. Statistical tests were performed with R 4.3.3 software.

## Results

### Subjective drug effects

**Fig. 1:**
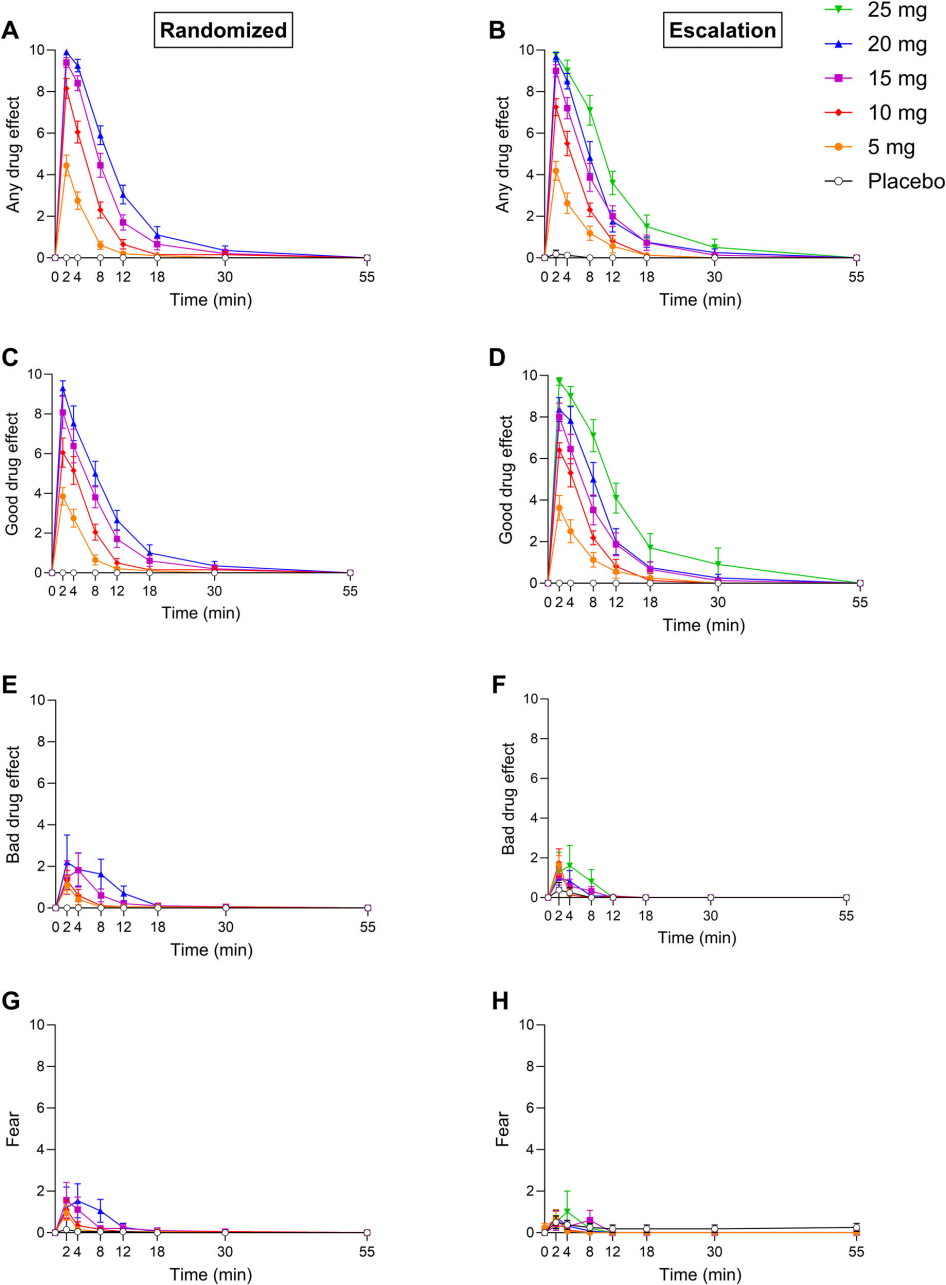
Acute subjective effects of *N,N*-dimethyltryptamine (DMT) over time. Bolus DMT doses rapidly induced acute subjective drug effects that peaked within 2 min and rapidly decreased and completely subsided within 12–30 min. The duration of effects dose-dependently increased. A ceiling effect was observed for peak drug effects starting at the 15 mg dose in the randomized arm. At equivalent dose levels, ratings of “any drug effect” were consistently lower in the dose-escalation study arm (**B**) compared with the randomized arm (**A**). Ratings of “good drug effect” were similar in both study arms (**C,** **D**), whereas ratings of “bad drug effect” (**E,** **F**) and “fear” (**G,** **H**) tended to be higher in the blinded randomized study arm compared with the dose-escalation arm at 15 and 20 mg. The data are expressed as the mean ± SEM in 20 healthy participants in the randomized study arm and in 16 (5 mg), 16 (10 mg), 15 (15 mg), 12 (20 mg), and 10 (25 mg) healthy participants in the dose-escalation study arm. The corresponding maximal responses and statistics are shown in Supplementary Tables S1 and S5.

Subjective effect scale ratings in both study arms are shown in Fig. 1. Statistics are summarized in *Supplementary* Table S2. In the randomized study arm, DMT dose-dependently elicited subjective responses that were significantly different from placebo, starting with the 5 mg dose (Fig. 1). All doses of DMT very rapidly induced strong subjective effects that peaked within the first 2 min after bolus administration and rapidly decreased and completely subsided within 12–30 min. A ceiling effect for the peak response was observed at the 15 mg dose (Fig. 1A). The effect duration increased with higher doses and increased further at the 20 mg dose, reflected by the significantly higher Area under the effect-time curve (AUEC) value for “any drug effect” following the 20 mg DMT dose compared with the 15 mg dose (Fig. 1A and Supplementary Table S2). On average, negative drug effect ratings were low compared with the much higher positive drug effect ratings. Nevertheless, the higher DMT doses of 15 and 20 mg induced more negative drug effects compared with the lower doses, reflected by higher “bad drug effect” and “fear” scores.

**Fig. 2:**
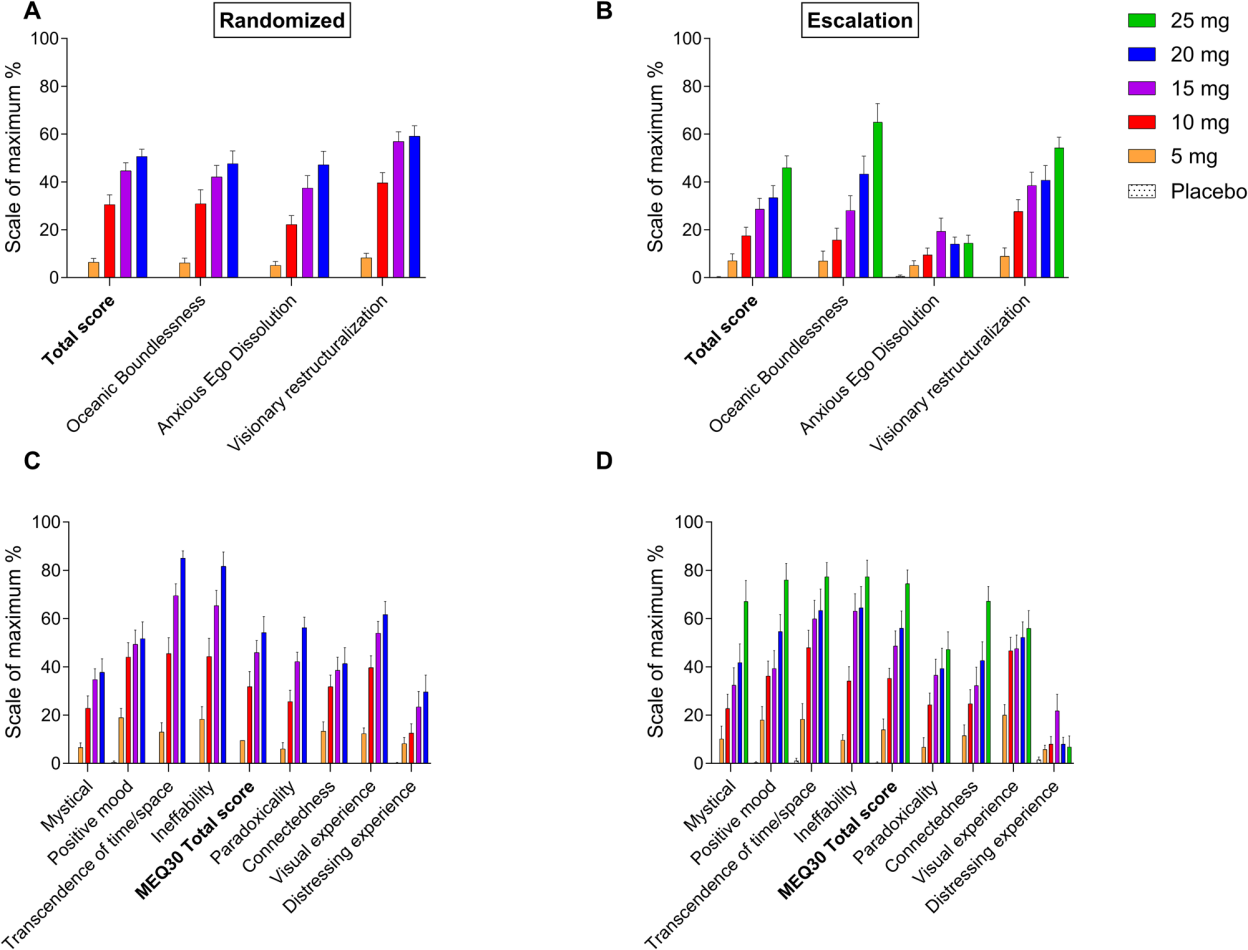
Acute psychedelic effects of *N,N*-dimethyltryptamine (DMT) rated 1 h after each administration. Acute psychedelic effects of DMT on the Altered States of Consciousness (3D-ASC) scale (**A,** **B**) and Psychedelic Experience Scale 48 (PES48) (**C,** **D**). DMT produced dose-dependent subjective psychedelic peak effects. A ceiling effect was observed for ratings on most subscales on the 3D-ASC and PES48 starting at 15 mg in the randomized arm. The 15 and 20 mg doses resulted in higher ratings of “anxious ego dissolution” on the 3D-ASC and “distressing experience” on the PES48 compared with the 5 and 10 mg doses (**A,** **C**). Conversely, no such ceiling effect was observed in the dose-escalation study arm, indicated by dose-dependent and linear increases in ratings on the 3D-ASC subscales (“oceanic boundlessness” and “visionary restructuralization”) and most of the PES48 subscales up to the maximal dose of 25 mg (**B,** **D**). At equivalent dose levels, ratings on the 3D-ASC and PES48 were consistently lower on all scales in the dose-escalation study arm compared with the randomized arm. This difference was most pronounced for negative drug effects, reflected by significantly lower ratings of “anxious ego dissolution” and “impaired control and cognition” on the 3D-ASC at 10, 15, and 20 mg in the dose-escalation study arm *vs*. randomized study arm. The data are expressed as the mean ± SEM in 20 healthy participants in the randomized study arm and in 16 (5 mg), 16 (10 mg), 15 (15 mg), 12 (20 mg), and 10 (25 mg) healthy participants in the dose-escalation study arm. The corresponding maximal responses and statistics are shown in Supplementary Tables S2 and S6.

Psychedelic effects on the 3D-ASC and PES48 and NDE-C are shown in Fig. 2 and *Supplementary* Fig. S1, respectively. Statistics are summarized in Supplementary Table S3. In the randomized arm, DMT produced pronounced psychedelic experiences at the 10, 15, and 20 mg doses (Fig. 2 and Supplementary Table S3). There was a ceiling effect on most subscale ratings on the 3D-ASC and PES48 at the 15 mg dose of DMT, with no significant differences between the 15 and 20 mg doses. The 5 mg dose of DMT elicited only mild psychedelic effects, with minimal increases across most 3D-ASC and PES48 subscales compared with placebo (Fig. 2 and Supplementary Table S3). Consistent with the subjective effect scale ratings, the 15 and 20 mg bolus doses resulted in stronger negative drug effects than the lower doses, reflected by significantly higher “anxious ego dissolution,” “anxiety,” and “impaired control and cognition” scores on the 3D-ASC and “distressing experience” scores on the PES48 (Figs. 2A and C and Supplementary Table S3). The lower tolerability of the 15 and 20 mg doses was also apparent in the dose-escalation study arm, in which three and two participants discontinued dose escalation after the 15 and 20 mg doses of DMT, respectively, whereas only one participant discontinued after the 10 mg dose.

### Comparison between the randomized and dose-escalation study arms

Subjective effects on the “any drug effect” scale were nominally higher across all dose conditions in the randomized study arm compared with the dose-escalation study arm (Fig. 1 and Supplementary Table S4). Ratings of “good drug effect” were similar between the study arms, whereas “bad drug effect” and “fear” scores were nominally higher in the randomized study arm compared with the dose-escalation study arm at the same doses of DMT (Fig. 1 and Supplementary Table S4). Consistent with these findings, participants in the randomized arm reported nominally higher scores on most subscales of the 3D-ASC and PES48 (Fig. 2 and Supplementary Table S5). Additionally, significant between-study-arm group differences were observed in the 3D-ASC total score and “anxious ego dissolution” and “impaired control and cognition” scores at the 10, 15, and 20 mg dose conditions. Moreover, positive drug effect increased overall dose-proportionally and linearly with no ceiling effect in the dose-escalation study arm, indicated by dose-proportional increases in ratings of “good drug effect” on the subjective effect scale (Fig. 1D), ratings of “oceanic boundlessness,” “blissful state,” “spiritual experience,” and “insightfulness” on the 3D-ASC (Fig. 2B and Supplementary Table S5), and ratings of “mystical” and “positive mood” on the PES48 (Fig. 2D and Supplementary Table S5) up to the maximal dose of 25 mg.

### Autonomic and adverse effects

Autonomic effects over time and related peak effects are shown in *Supplementary* Fig. S2 and Supplementary Table S2, respectively. DMT dose-dependently increased systolic and diastolic blood pressure. Acute adverse effects during the entire study session are presented in Supplementary Table S6. Frequent complaints included headache, impaired concentration, feeling of weakness, and palpitations. One participant required follow-up care by the study psychiatrist because of the onset of anxiety and panic attacks that the participant retrospectively reported as beginning shortly after the study session. These symptoms resolved within 2 months but reappeared 3 months later, and the participant has since been treated with outpatient psychotherapy and escitalopram.

### Pharmacokinetics and pharmacokinetic-pharmacodynamic relationship

**Fig. 3:**
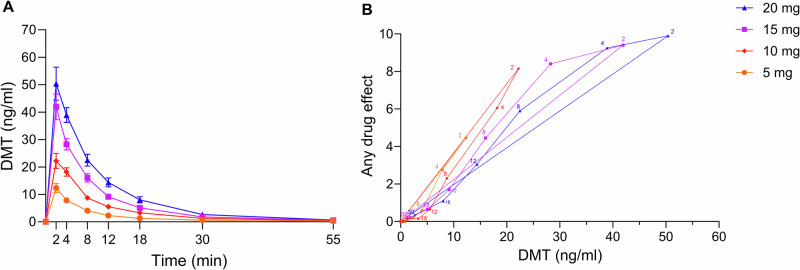
Pharmacokinetics and pharmacokinetic-pharmacodynamic relationship of *N,N*-dimethyltryptamine (DMT) in the randomized study arm. **A** Plasma DMT concentrations increased proportionally with increasing doses. Plasma concentrations increased rapidly within the first 2 min across all doses. DMT concentrations then rapidly declined within 30 min. The corresponding pharmacokinetic parameters are shown in Table 1. Individual concentrations are shown in *Supplementary* Fig. S4. **B** Subjective effects closely mirrored plasma DMT concentrations at 5 and 10 mg. At 15 and 20 mg, subjective effects remained near peak levels at 4 min after bolus administration despite declining plasma concentrations that showed counterclockwise hysteresis and some lag in the response relative to plasma concentrations. Three was no evidence of acute pharmacological tolerance. Plasma concentration values are expressed as the mean, and responses are expressed as the mean on the subjective effect “any drug effect” scale (*n* = 19). The time of sampling is indicated (in minutes) next to each data point.

Table 1 and Supplementary Table S7 show pharmacokinetic parameters of DMT based on noncompartmental analyses of the randomized and dose-escalation study arms, respectively. Concentration-time curves of DMT are shown in Fig. 3 and *Supplementary* Fig. S3. Individual concentration-time curves are shown in *Supplementary* Figs. S4 and S5. Concentration-time curves of DMT on a semilogarithmic plot are shown in Supplementary Fig. S6. Concentration-time curves of the main metabolites indole-3-acetic acid and DMT-*N*-oxide are shown in Supplementary Fig. S7. Plasma DMT concentrations increased dose-proportionally (Table 1 and Fig. 3). In the randomized arm, the mean maximum plasma concentrations (C_max_) for the 5, 10, 15, and 20 mg doses were 11, 20, 37, and 45 ng/ml, respectively, and were reached (T_max_) after 2.3, 2.7, 2.4, and 2.5 min, respectively. After an initial distribution phase of 2–4 min, the mean plasma half-life of DMT was 6.1–6.8 min (Table 1). From 30–55 min, a longer half-life was observed based on inspection of the semilogarithmic plots (Supplementary Fig. S6). Because of limited pharmacokinetic sampling in the period between 30 and 55 min, the longer half-life could not be reliably estimated (Table 1).

**Table 1:** Pharmacokinetic parameters for DMT based on non-compartmental analyses [geometric mean (95% CI), range] in the double-blinded and randomized treatment study arm.

Dose	5 mg	10 mg	15 mg	20 mg
Number of participants	19*	19*	19*	19*
C_max_ (ng/ml)	11 (8.2–14)	20 (16–26)	37 (30–46)	45 (37–55)
	3.5–30	5.5–52	13–76	28–121
T_max_ (min)	2.3 (1.9–2.8)	2.7 (2.3–3.2)	2.4 (2.1–2.9)	2.5 (2.1–2.9)
	2–8	2–4	2–5	2–4
t_1/2_ (min)	6.7 (5.6–8.1)	6.1 (5.0–7.4)	6.5 (5.7–7.4)	6.8 (6.0–7.6)
	3.6–15	2.6–11	4.1–12	4.2–11
CL (L/min)	48 (38–60)	48 (38–61)	39 (33–47)	41 (34–48)
	21–105	23–119	23–73	23–61
V_z_ (L)	465 (390–555)	423 (329–545)	370 (314–437)	397 (332–474)
	232–974	117–1592	218–670	195–642
AUC_∞_ (ng*min/ml)	104 (82–130)	209 (165–264)	380 (317–456)	492 (414–585)
	48–239	84–436	204–640	330–882

*AUC* area under the plasma concentration-time curve, *AUC*_*∞*_ AUC from time zero to infinity, *CL* apparent total clearance *C*_*max*_ maximum observed plasma concentration *t*_*1/2*_ plasma elimination half-life, *T*_*max*_ time to reach C_max_, *95%CI*, 95% confidence interval, *V*_*z*_, apparent volume of distribution, *n* = 19.

^*^One participant was excluded from analysis due to missing plasma samples.

The DMT concentration-response relationship over time is shown in Fig. 3 and Supplementary Fig. S3 for the randomized and dose-escalation arms, respectively. In the randomized arm, subjective effects closely mirrored plasma DMT concentrations at 5 and 10 mg, with no hysteresis and thus no evidence of acute pharmacological tolerance. At 15 and 20 mg, subjective effects remained near peak levels at 4 min after bolus administration despite declining plasma concentrations, indicating a ceiling effect at these doses. In contrast, in the dose-escalation arm, a ceiling effect was observed only at the 20 and 25 mg doses. “Any drug effect” ratings were similar at equivalent doses with repeated hourly dosing (Supplementary Table S8), indicating no tolerance to acute effects of repeated bolus DMT administrations.

### Blinding

Data on the participants’ retrospective identification of the DMT dose conditions are shown in Supplementary Table S9. Most conditions were correctly identified by the participants at the end-of-study visit once all conditions could be compared. However, when asked after each administration, the 5, 10, 15, and 20 mg doses were misclassified by two (13%), nine (56%), five (31%), and seven (44%) participants, respectively. In most of these cases, the conditions were mistaken as the next higher or lower dose.

## Discussion

In the present study, we characterized pharmacokinetics and acute dose-dependent effects of intravenous DMT administration as bolus doses in healthy participants.

Subjective peak effects of DMT were reached rapidly within 1–3 min. There was a ceiling effect for the peak subjective response intensity, with high effect ratings reached at the 15 mg dose. Subjective effects of the 5 and 10 mg doses declined rapidly after peaking at 2 min after bolus administration but remained close to peak intensity for approximately 4 min at the 15–25 mg doses. Thus, the effect duration was dose-dependent, ranging from 12–30 min across the 5–20 mg doses. The present findings are consistent with prior studies, in which subjective peak effects were reached within 1–3 min, and effect durations were up to 20–30 min at high doses of intravenous DMT administration [3, 6, 7, 12, 13, 26]. One study reported delayed peak effects that occurred 5–10 min after bolus administration [8], likely attributed to the smaller injection volume (1 ml) that was used. In contrast, both our studies [3] and others [6, 7, 12, 13, 26] likely achieved more rapid drug availability in the circulation by using a larger injection volume (20 ml), as in our case, or a saline flush following the bolus.

Intravenous bolus DMT administration in the present study produced very intense effects, including moderate anxiety, at the higher doses. Consistent with a previous study, intravenous bolus doses were less well tolerated than continuous infusions of DMT, primarily because of the overwhelming intensity and rapid onset of effects at doses of 15 and 25 mg [3]. Participants in the present study described similar experiences, characterizing the initial drug effects as simultaneously intense, good, bad, and anxiety-inducing, particularly at the higher doses of 15 and 20 mg. Notably, despite these often overwhelming effects, all of the participants in the randomized study arm of the present study completed the full study, which involved multiple bolus administrations of DMT within a single day, each separated by only 1 h. Nevertheless, the intense subjective effects were experienced as positive overall, indicating acceptable tolerability. These findings are consistent with other studies that used intravenous bolus or inhalation DMT administration [6–8, 10, 12, 13].

The present study also determined pharmacokinetic parameters of DMT across a wide dose range of intravenous bolus doses. DMT was cleared very rapidly from plasma, with an initial half-life of 6–7 min during the first 20–30 min post-administration. Plasma concentrations then declined more slowly until 55 min. However, because of the limited sampling during this later phase, a second half-life could not be reliably estimated. These pharmacokinetic findings are consistent with our previous studies that described an early half-life of DMT of 5–7 min during the first 20 min and a late half-life of 15–19 min following the cessation of continuous DMT infusion after 90 and 120 min, respectively [3, 4]. Another study reported an overall longer mean plasma half-life of 9–12 min for DMT but did not differentiate between the early short and later long plasma half-lives [5]. We observed no acute pharmacological tolerance to acute effects of bolus DMT doses in the present study. Specifically, there was no clockwise hysteresis curve in the concentration-effect relationship plot, indicating no tolerance during the response to a single dose. Additionally, there was no decline in the response to multiple DMT dose administrations, and no order effects were detected. These findings are consistent with early studies by Strassman, who reported no tolerance to subjective effects in a study that involved closely spaced bolus DMT doses of 0.3 mg/kg [12]. In contrast, moderate acute tolerance has been described with continuous intravenous DMT administration [3, 4]. This phenomenon, in which continuous infusions induce tolerance but intermittent dosing does not, has been observed for other substances, including nitroglycerin [27], benzodiazepines, and opioids [28]. Thus, the presence or absence of tolerance depends on the type of administration (intermittent/short-lasting *vs*. continuous/longer-lasting) rather than a property of DMT itself. Possible mechanisms that could explain this differential tolerance include receptor desensitization, internalization, and downstream adaptations in signaling efficiency that are caused by sustained agonist exposure with continuous administration [29].

In the double-blinded randomized study arm, blinding was relatively well maintained in the present study because of the use of different doses. Typically, doses of 10–20 mg were mistaken as the next higher or lower dose. Conversely, placebo and the 5 mg dose conditions were guessed correctly in > 80% of cases. The relatively high accuracy in identifying placebo and the low-dose condition immediately after the session (i.e., before receiving further doses) highlights the difficulty of maintaining blinding with psychoactive compounds. The findings suggest that even an active placebo, such as the 5 mg DMT dose condition in this context, may not always be sufficient to maintain adequate blinding against high doses.

The present study included an additional dose-escalation study arm. In the open-label dose-escalation study arm, 10 of 16 participants (63%) increased dosing up to the maximum dose of 25 mg, including three who had never had a psychedelic previously. One participant stopped after the 10 mg dose, three stopped after the 15 mg dose, and two stopped after the 20 mg dose. Notably, at equivalent dose levels, participants in the dose-escalation arm reported lower ratings of acute subjective effects compared to the randomized arm, with ratings of negative effects nearly 50% lower.

Moreover, a ceiling effect for the peak subjective effect response emerged only at 20 mg or higher, whereas in the randomized arm, a ceiling effect was already observed at 15 mg. Consistent with this, overall peak psychedelic effect ratings (total 3D-ASC scores) were 50% lower on scales that reflected positive effects, such as “oceanic boundlessness,” and 60% lower on scales that reflected negative effects, such as “anxious ego dissolution,” in the dose-escalation arm compared with the randomized study arm. Psychedelically experienced individuals could be expected to report weaker subjective effects compared with psychedelically naive individuals [30, 31]. However, participants in the dose-escalation arm were less experienced than those in the randomized arm, but they consistently rated their experiences as less intense. The open-label dose-escalation design likely allowed participants to be more prepared for the gradually increasing psychedelic effects, whereas participants in the randomized arm might have been overwhelmed and surprised by the sudden onset and intensity of effects of the randomly ordered doses and not knowing whether a next dose might even be higher. Additionally, expectancy effects—in this case, knowing the dose and even being in control of the dose escalation—may have provided participants with a sense of control and reassurance before each administration, thus minimizing especially negative effects. Similarly, in a previous study, in which participants were allowed to self-titrate the dose rate to their individually preferred level during a continuous DMT infusion, negative effects were minimal, despite many participants increasing the dose rate to induce strong psychedelic effects [4]. These findings highlight the importance of context in psychedelic research [32]. In particular, the findings illustrate the role of open-label *vs*. blinded administration in moderating the subjective psychedelic experience [33, 34]. Previous trials demonstrated the good tolerability of psychedelics in healthy participants [3, 4, 35–39] and patients [35, 36, 40–45] who implement dose titration, which is commonly practiced for many medications and may further enhance tolerability. This approach is simple for intravenous DMT administration, in which bolus doses can be steadily increased as in the present study within a session or adjusted during a continuous intravenous infusion [4]. DMT that is administered as an intravenous bolus or via inhalation produced rapid-acting antidepressant effects in initial studies [8, 15]. The present study indicates that the tolerability of DMT could be enhanced by escalation dosing, and the findings may optimize dosing in future clinical studies.

The present study has several notable strengths. Four different doses of DMT were administered in a cross-over design and compared with placebo under double-blind conditions in a controlled laboratory setting. Thus, we were able to accurately describe the dose-response relationship of DMT bolus doses across a wide dose range. We also included equal numbers of male and female participants and used internationally established standardized and validated psychometric outcome measures. Moreover, we tested a dose-escalation regimen that could be used in the future in patients to enhance tolerability and individualize treatment with DMT. Lastly, the direct comparison between the double-blind, randomized arm and open-label, dose-escalation arm allowed the evaluation of how design, dosing, blinding, and associated expectancy effects can influence subjective responses.

Notwithstanding these strengths, the present study also has limitations. First, participants with lower tolerability of DMT were more likely to drop out early during dose escalation, potentially introducing a bias toward more positive effects at higher doses in that arm. However, all but one participant reached the 15 mg dose, and 12 of 16 participants (75%) received the 20 mg dose. Therefore, the direct comparison between the randomized and dose-escalation arms can be considered valid at least up to the 15 mg dose. Second, participants were not randomly assigned to the two study arms but were allowed to choose their preferred arm. This self-selection introduces a potential bias; one would expect more cautious or less psychedelically experienced individuals to opt for the dose-escalation arm, which was indeed the case. Moreover, the study was powered for within-subject dose comparisons within each study arm rather than between-subject differences across the two study arms, making this comparison between study arms exploratory. Lastly, the study used a highly controlled setting and included only healthy participants. Thus, subjects in different environments and patients with psychiatric disorders may respond differently to DMT.

## Conclusion

Bolus DMT doses exhibited dose-proportional pharmacokinetics and produced dose-dependent subjective peak effects that reached a ceiling at doses > 15 mg. DMT showed better tolerability when dosed open-label and with gradual dose escalation rather than when the doses were double-blinded and random. The present findings may support dosing in future clinical studies and therapeutic use.

## Supplementary information

Supplementary_material_DMT-BDR
