# Supplementary material for: Dose-dependent pharmacokinetics and acute effects of intravenous bolus N,N-dimethyltryptamine: double-blind, randomized versus open-label dose-escalation administration study in healthy participants
Source: Transl Psychiatry. 2026 Mar 27;16:213. doi: 10.1038/s41398-026-03987-7 (PMC13039932; doi:10.1038/s41398-026-03987-7)
Supplement: Supplementary file 1 — Supplementary_material_DMT-BDR [file 41398_2026_3987_MOESM1_ESM.pdf]

Supplement

Methods

Subjective effect scales (SES)

Participants were asked by the investigator to repeatedly rate their subjective effects verbally on four single-item Likert scales from 0 to 10 for: “any drug effect”, “good drug effect”, “bad drug effect”, and “fear”. Intervals were 0, 2, 4, 8, 12, 18, 30, and 55 minutes after drug administration. A similar method was used previously to assess DMT effects [1-3] and is less demanding than completing self-rated single-item visual rating scales (VAS) in written form [4] and therefore interferes only minimally with the subjective experience. The SES “any drug effect” is an overall effect measure to characterize the effect intensity and time course. The SES “good drug effect” is an overall measure of effects subjectively considered positive. The SES “bad drug effect” is an overall measure of any negative effects and related to “fear”. SES scales were assessed each time DMT blood concentrations were measured to allow for pharmacokinetic-pharmacodynamic modeling.

3 Dimensions of Altered States of Consciousness (3D-ASC) scale

A short version of the Altered States of Consciousness (*3D-ASC*) Scale comprising 42 items (instead of 66 items) [5,6] was used as the primary outcome measure and was administered 30 minutes after drug administration to retrospectively rate peak drug effects. The shorter version conserves the underlying factor structure. Thus, the same three subscales/dimensions [5] and 11 lower-order scales [6] can be calculated. The 3D-ASC dimension “Oceanic Boundlessness” (16 items) measures derealization and depersonalization associated with positive emotional states, ranging from heightened mood to euphoric exaltation. The corresponding lower-order scales include “experience of unity,” “spiritual experience,” “blissful state,” “insightfulness,” and “disembodiment.” The dimension “Anxious Ego Dissolution” (13 items) summarizes ego-disintegration and loss of self-control phenomena associated with anxiety. The corresponding lower-order scales include “impaired control of cognition” and “anxiety.” The dimension “Visionary Restructuralization” (12 items) consists of the lower-order scales “complex imagery,” “elementary imagery,” “audio-visual synesthesia,” and “changed meaning of percepts.” The total 3D-ASC score is the total of the three main dimensions “Oceanic Boundlessness”, “Anxious Ego-Dissolution”, and “Visionary Restructuralization” and can be used as a measure of the overall intensity of the alteration of the mind [7]. The scale is well-validated in German [5] and many other languages and widely used to characterize the subjective effects of various psychedelic drugs. In particular, the scale has been used by most research groups to psychometrically assess LSD effects [8-13]. Furthermore, acute ratings on the 3D-ASC after administration of psilocybin have been used to predict long-term effects of

psychedelic treatments in patients [14,15]. Ratings on the 3D-ASC have been shown to closely correlate with ratings on the Mystical Effects Questionnaire (MEQ, see below) [7] which is primarily used by research groups in the US [15].

Psychedelic Experience Scale (PES48)

The Psychedelic Experience Scale (PES48) was administered 30 min after drug administration to retrospectively rate peak subjective effects. The PES48 [16] is a revalidation of the original 100-item States of Consciousness Questionnaire (SOCQ) [7,17] and includes the 30-item Mystical Effects Questionnaire (MEQ30) [18], as well as four additional subscales. The MEQ30 consists of the subscales “mystical experience”, “positive mood”, “transcendence of time and space”, and “ineffability”. Their sum provides the MEQ30 total score, which reflects the overall intensity of the mystical experience. The MEQ30 has been used in numerous experimental trials with LSD [9,11,19-22]. Additional subscales of the PES48 assess “paradoxicality” and “connectedness,” “visual experience” and “distressing experience”. The published German version was used [7,16]. It should be noted that participants completed only the 48 items relevant for the PES48 and not the full 100 items of the original SOCQ questionnaire (no distractor items).

NDE-C Scale

The Near-Death Experience Content (NDE-C) scale is a modified version of the NDE-scale [23]. It assesses 20 items rated on a 5-point Likert scale. It is a tool to assess dimensions of near-death experience and has been psychometrically validated [23]. We used a slightly modified, non-validated version translated into German. Specifically, we added nine new items assessing dimensions of fear, non-existence, loneliness, void, borderline experience, disintegration and rebirth in more detail.

Results

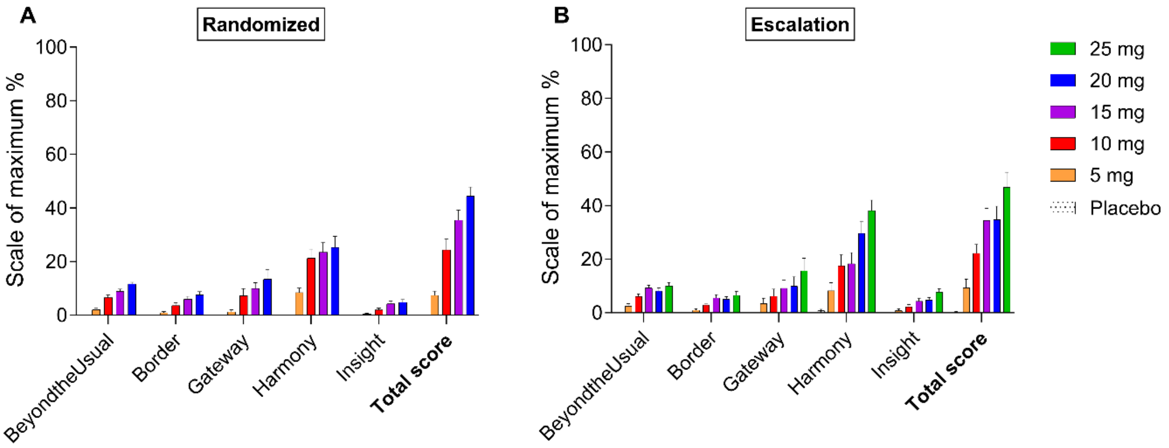

Figure S1. Near-death experience-like effects of N,N-Dimethyltryptamine (DMT) in the randomized **(A)** and dose-escalation **(B)** study arm on the NDE-C scale. The data are expressed as the mean ± SEM in 20 healthy participants in the randomized study arm and in 16 (5 mg), 16 (10 mg), 15 (15 mg), 12 (20 mg), and 10 (25 mg) healthy participants in the dose-escalation study arm.

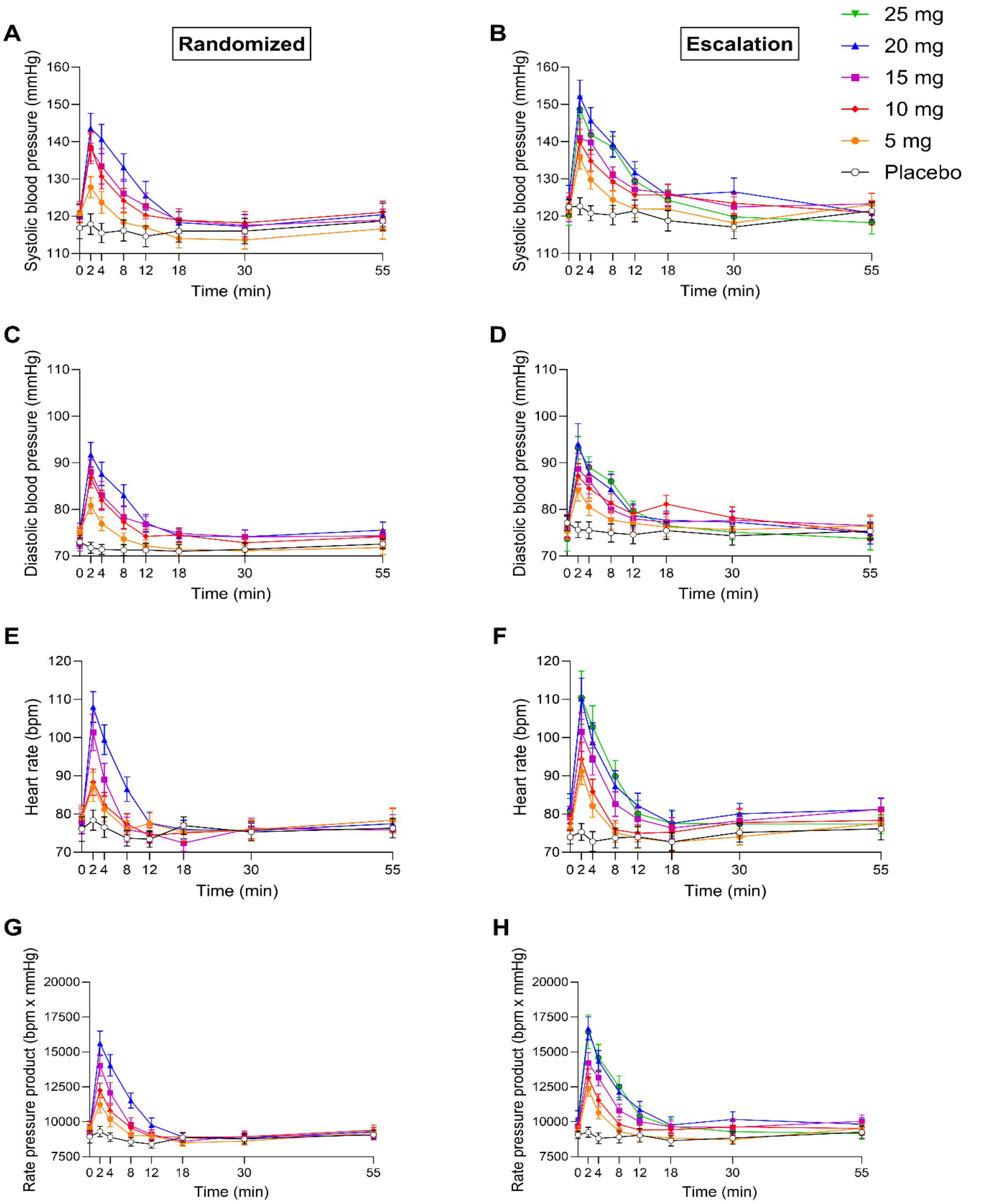

Figure S2. Acute autonomic effects of *N,N*-dimethyltryptamine (DMT) over time in the randomized and dose-escalation study arm. DMT dose-dependently increased blood pressure and heart rate compared with placebo. Effects normalized within 20–30 minutes after bolus administration. The data are expressed as the mean ± SEM in 20 healthy participants in the randomized study arm and in 16 (5 mg), 16 (10 mg), 15 (15 mg), 12 (20 mg), and 10 (25 mg) healthy participants in the dose-escalation study arm. Maximal effects and statistics are shown in *Supplementary Table S2*.

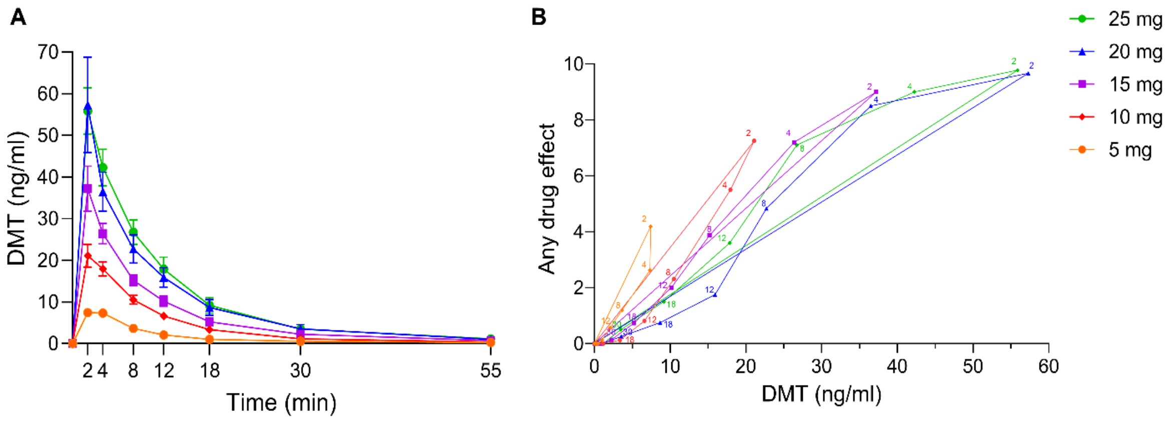

Figure S3. Pharmacokinetics and pharmacokinetic-pharmacodynamic relationship of *N,N*-dimethyltryptamine (DMT). **(A)** Plasma DMT concentration-time curves for 5, 10, 15, 20, and 25 mg doses in the dose-escalation study arm. Plasma DMT concentrations increased proportionally with increasing doses. Plasma concentrations peaked within the first two minutes across all doses. Then, DMT concentrations rapidly declined within 30 minutes. Plasma concentration values are expressed as the mean ± SEM in 16 (5 mg), 16 (10 mg), 15 (15 mg), 12 (20 mg), and 10 (25 mg) healthy participants. The corresponding pharmacokinetic parameters are shown in *Supplementary Table S7*. Individual concentrations are shown in *Supplementary Figure S5*. **(B)** Plasma concentration-response relationships for 5, 10, 15, 20 and 25 mg doses of DMT in the dose-escalation study arm. Plasma concentration values are expressed as the mean, and responses are expressed as the mean on the subjective effect scale of “any drug effect” in 16 (5 mg), 16 (10 mg), 15 (15 mg), 12 (20 mg), and 10 (25 mg) healthy participants. The time of sampling (in minutes) is indicated next to each data point. Subjective effects closely mirrored DMT plasma concentrations at 5,10 and 15 mg, indicating no delay in effect or tolerance. At 20 and 25 mg, subjective effects remained near peak levels at 4 minutes after bolus administration despite declining plasma concentrations, indicating a ceiling effect at these doses.

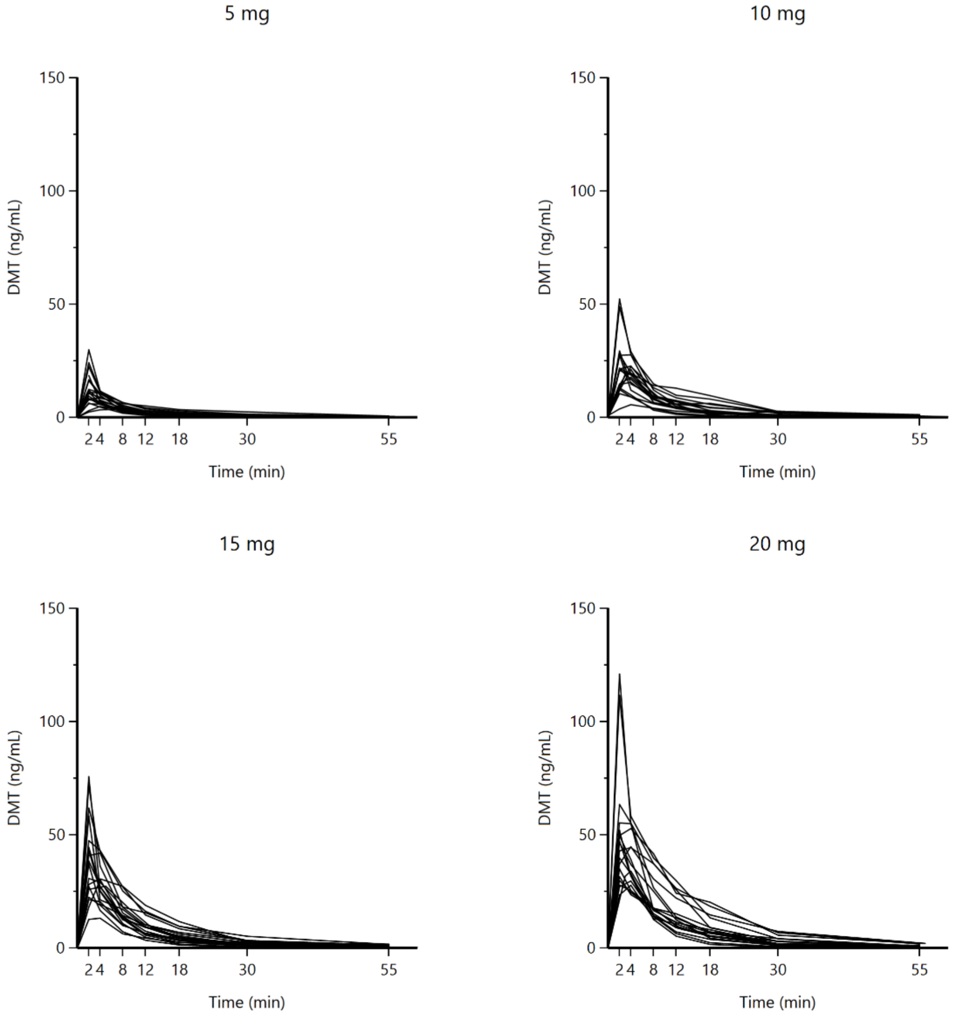

Figure S4. Individual plasma concentrations of *N,N*-dimethyltryptamine (DMT) over time in the randomized study arm. Data are shown for 19 healthy participants. One participant was excluded from analysis due to missing plasma data.

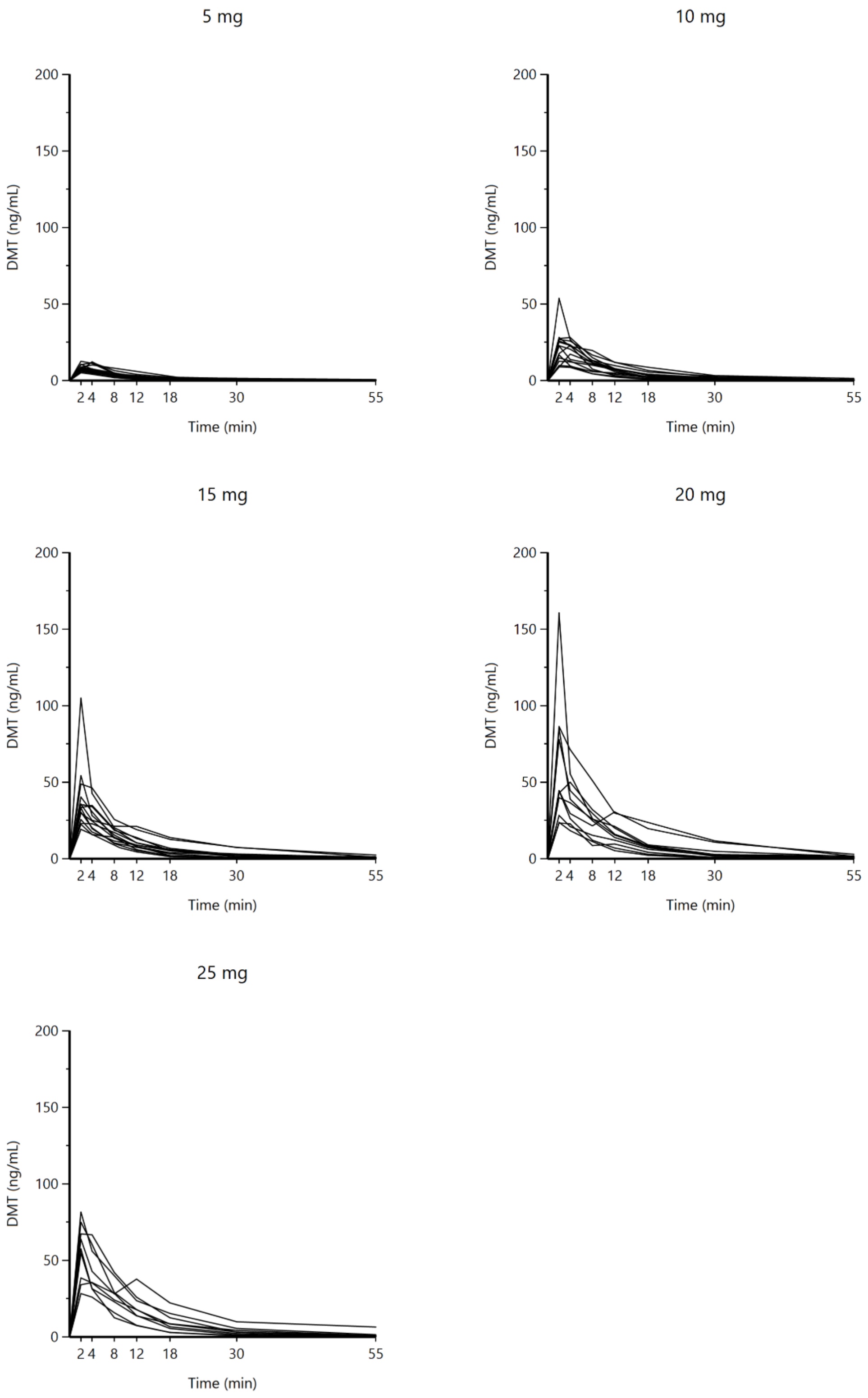

Figure S5. Individual plasma concentrations of *N,N*-dimethyltryptamine (DMT) over time in the dose-escalation study arm. Data are shown for 15 (5 mg), 15 (10 mg), 14 (15 mg), 11 (20 mg), and 9 (25 mg) healthy participants. One participant was excluded from analysis due to missing plasma data.

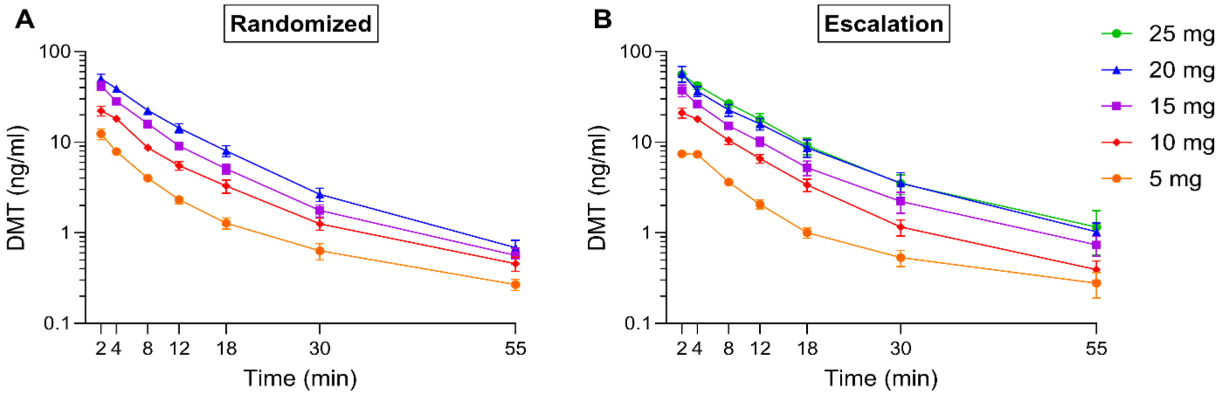

Figure S6. Plasma concentrations of *N,N*-dimethyltryptamine (DMT) over time on a semilogarithmic plot in the randomized **(A)** and dose-escalation **(B)** study arm. There was an early fast elimination within the first 30 minutes after bolus administration, with a half-life of 6–7 minutes. Visual inspection suggests a longer half-life between 30 until 55 minutes; however, reliable estimation was not possible due to limited sampling during this period. The data are expressed as the mean ± SEM in 20 healthy participants in the randomized study arm and in 16 (5 mg), 16 (10 mg), 15 (15 mg), 12 (20 mg), and 10 (25 mg) healthy participants in the dose-escalation study arm.

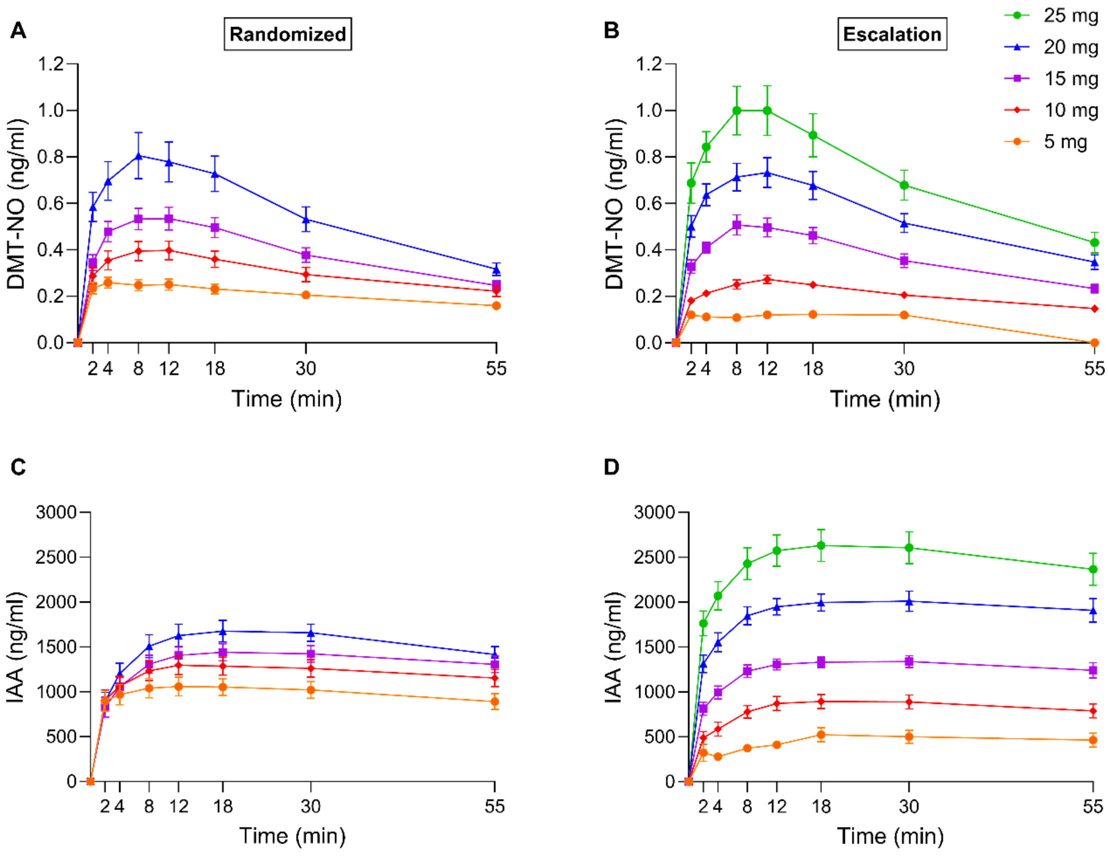

Figure S7. Plasma concentrations of DMT-*N*-oxide (DMT-NO) **(A,B)** and indole-3-acetic acid (IAA) **(C,D)** over time in the randomized and dose-escalation study arm. The data are expressed the mean ± SEM in 20 healthy participants in the randomized study arm and in 16 (5 mg), 16 (10 mg), 15 (15 mg), 12 (20 mg), and 10 (25 mg) healthy participants in the dose-escalation study arm.

Table S1. Baseline Characteristics

**Study arm**		**Randomized (n = 20)**	**Escalated (n = 16)**
**General Characteristics**			
Female	n (%)	10 (50)	8 (50)
Psychedelic-naïve	n (%)	4 (20)	6 (37)
Age	mean (sd)	34 (9.2)	39 (13.0)
Weight	mean (sd)	67 (11.6)	70 (13.7)
BMI	mean (sd)	22 (2.4)	23 (2.9)
**Lifetime substance experiences***			
Psychedelics			
LSD	n (range)	8 (1–5)	7 (1–3)
Psilocybin	n (range)	14 (1–4)	5 (1–3)
DMT (including ayahuasca)	n (range)	5 (1–4)	3 (1–5)
Mescaline	n (range)	2 (1)	2 (1–5)
2C–B	n (range)	2 (1)	1 (1)
MDMA	n (range)	8 (2–30)	7 (1–5)
Stimulants			
Amphetamine	n (range)	3 (1–40)	2 (3–5)
Cocaine	n (range)	5 (1–5)	1 (1)
Methylphenidate	n (range)	0 (0)	1 (3)
Further substances			
Ketamine	n (range)	6 (1–20)	2 (3–4)
Opioids	n (range)	2 (1–2)	0 (0–0)
**Personality traits (NEO-FFI)**			
Neuroticism	mean (sd)	1.25 (0.65)	1.54 (0.68)
Extraversion	mean (sd)	2.48 (0.47)	2.52 (0.51)
Openness	mean (sd)	3.06 (0.49)	2.82 (0.43)
Agreeableness	mean (sd)	3.19 (0.5)	3.07 (0.47)
Conscientiousness	mean (sd)	2.91 (0.59)	2.72 (0.46)

DMT; N,N-Dimethyltryptamine, LSD; Lysergic acid diethylamide, MDMA; 3,4-methylenedioxymethamphetamine, 2C-B; 4-bromo-2,5-dimethoxyphenylethylamine, sd; standard deviation, NEO-FFI; NEO-Five-Factor Inventory

* "n" refers to the number of participants and "range" to the number of reported uses among those participants

Table S2. Mean values and statistics for the acute subjective and autonomic effects of DMT and placebo in the double-blind and randomized treatment study arm

		**Placebo**	**5 mg**	**10 mg**	**15 mg**	**20 mg**		Pla - 5 mg	Pla - 10 mg	Pla - 15 mg	Pla - 20 mg	5 mg - 10 mg	5 mg - 15 mg	5 mg - 20 mg	10 mg - 15 mg	10 mg - 20 mg	15 mg - 20 mg
		mean ± SEM	mean ± SEM	mean ± SEM	mean ± SEM	mean ± SEM	*p* =										
Subjective Effects																	
Any drug effect	ΔE_{max}	0.0 ± 0.0	4.5 ± 0.5	8.2 ± 0.5	9.5 ± 0.2	9.9 ± 0.1	< 0.001	***	***	***	***	***	***	***	*	***	ns
	AUEC	0.0 ± 0.0	21 ± 3.5	51 ± 5.7	80 ± 8.8	103 ± 10	< 0.001	*	***	***	***	**	***	***	**	***	*
	T_{max} (min)	NA	2.0 ± 0.0	2.0 ± 0.0	2.1 ± 0.1	2.0 ± 0.0	ns	NA	NA	NA	NA	ns	ns	ns	ns	ns	ns
Good drug effect	ΔE_{max}	0.0 ± 0.0	4.1 ± 0.4	6.5 ± 0.71	7.3 ± 0.7	7.9 ± 0.7	< 0.001	***	***	***	***	**	***	***	ns	ns	ns
	AUEC	0.0 ± 0.0	20 ± 3.5	42 ± 6.6	63 ± 9.9	80 ± 12	< 0.001	ns	***	***	***	ns	***	***	ns	***	ns
Bad drug effect	ΔE_{max}	0.0 ± 0.0	1.1 ± 0.4	1.6 ± 0.5	2.4 ± 0.7	2.7 ± 0.9	< 0.01	ns	ns	*	**	ns	ns	ns	ns	ns	ns
	AUEC	0.0 ± 0.0	4.3 ± 1.9	6.3 ± 2.5	13 ± 4.2	20 ± 6.4	< 0.001	ns	ns	*	***	ns	ns	*	ns	*	ns
Fear	ΔE_{max}	0.2 ± 0.1	1.0 ± 0.4	1.3 ± 0.4	2.0 ± 0.7	1.9 ± 0.8	< 0.05	ns	ns	*	ns	ns	ns	ns	ns	ns	ns
	AUEC	0.9 ± 0.8	3.3 ± 1.3	5.3 ± 2.6	10 ± 3.8	13 ± 5.6	< 0.01	ns	ns	ns	*	ns	ns	ns	ns	ns	ns
Autonomic Effects																	
Systolic blood pressure (mmHg)	E_{max}	124 ± 3.0	130 ± 2.8	138 ± 3.7	140 ± 3.9	145 ± 3.5	< 0.001	ns	***	***	***	*	***	***	ns	*	ns
Diastolic blood pressure (mmHg)	E_{max}	77 ± 1.2	82 ± 1.6	87 ± 1.9	89 ± 2.4	92 ± 2.3	< 0.001	ns	***	***	***	*	***	***	ns	*	ns
Heart rate (beats/min)	E_{max}	84 ± 2.7	91 ± 3.4	92 ± 3.3	102 ± 4.5	108 ± 3.7	< 0.001	ns	ns	***	***	ns	**	***	*	***	ns
RPP (mmHg x bpm)	E_{max}	10121 ± 368	11609 ± 533	12346 ± 447	14066 ± 743	15678 ± 744	< 0.001	ns	**	***	***	ns	**	***	*	***	ns

*p<0.05, **p<0.01, ***p<0.001; ns, not significant; AUEC, Area under the effect-time curve; ΔEmax, maximal effect difference from baseline; Pla, Placebo; RPP, Rate pressure product; Tmax, time to reach Emax.

Table S3. Mean values and statistics for the acute subjective effects of the four DMT conditions and Placebo on the 3 Dimensions of Altered States of Consciousness (3D-ASC) Scale, the Psychedelic Experience Scale (PES48), and Near Death Experience Scale (NDE-C)

	**Placebo**	**5 mg**	**10 mg**	**15 mg**	**20 mg**		Pla - 5 mg	Pla - 10 mg	Pla - 15 mg	Pla - 20 mg	5 - 10 mg	5 - 15 mg	5 - 20 mg	10 - 15 mg	10 - 20 mg	15 - 20 mg
	mean ± SEM (% score)	mean ± SEM (% score)	mean ± SEM (% score)	mean ± SEM (% score)	mean ± SEM (% score)	*p*										
Altered States of Consciousness (3D-ASC) Scale																
OAV total Score	0 ± 0	6.5 ± 1.6	31 ± 4.2	45 ± 3.5	51 ± 3.2	< 0.001	ns	***	***	***	***	***	***	***	***	ns
Oceanic boundlessness	0 ± 0	6.2 ± 2.0	31 ± 6.1	42 ± 5.0	48 ± 5.6	< 0.001	ns	***	***	***	***	***	***	ns	*	ns
Anxious ego-dissolution	0 ± 0	5.2 ± 1.6	22 ± 3.9	37 ± 5.4	47 ± 5.8	< 0.001	ns	***	***	***	**	***	***	*	***	ns
Visionary restructuralization	0 ± 0	8.3 ± 2.0	40 ± 4.4	57 ± 4.2	59 ± 4.6	< 0.001	ns	***	***	***	***	***	***	***	***	ns
Experience of unity	0 ± 0	4.1 ± 1.8	30 ± 6.7	48 ± 6.5	54 ± 7.4	< 0.001	ns	***	***	***	**	***	***	ns	**	ns
Spiritual experience	0 ± 0	4.4 ± 1.4	22 ± 5.6	29 ± 5.4	33 ± 5.2	< 0.001	ns	***	***	***	*	***	***	ns	ns	ns
Blissful state	0 ± 0	14 ± 3.9	41 ± 7.6	44 ± 7.5	41 ± 8.5	< 0.001	ns	***	***	***	**	**	*	ns	ns	ns
Insightfulness	0 ± 0	5.0 ± 3.6	13 ± 3.7	19 ± 4.8	21 ± 5.2	< 0.001	ns	ns	**	***	ns	*	*	ns	ns	ns
Disembodiment	0 ± 0	3.9 ± 1.7	41 ± 7.6	58 ± 6.3	74 ± 5.9	< 0.001	ns	***	***	***	***	***	***	ns	***	ns
Impaired control and cognition	0 ± 0	6.8 ± 2.2	31 ± 5	48 ± 5.5	61 ± 5.4	< 0.001	ns	***	***	***	***	***	***	*	***	ns
Anxiety	0 ± 0	3.3 ± 1.3	12 ± 4.3	25 ± 7.3	31 ± 7.8	< 0.001	ns	ns	***	***	ns	**	***	ns	*	ns
Complex imagery	0 ± 0	6.4 ± 2.6	42 ± 8.4	64 ± 7.1	65 ± 6.7	< 0.001	ns	***	***	***	***	***	***	*	**	ns
Elementary imagery	0 ± 0	19 ± 5.1	66 ± 6.1	80 ± 4.2	83 ± 5.5	< 0.001	*	***	***	***	***	***	***	ns	ns	ns
Audio-visual synesthesia	0 ± 0	7.0 ± 2.8	42 ± 7.2	65 ± 6.8	67 ± 8	< 0.001	ns	***	***	***	***	***	***	**	**	ns
Changed meaning of percepts	0 ± 0	1.8 ± 0.7	20 ± 5.2	32 ± 6.7	37 ± 6.1	< 0.001	ns	**	***	***	*	***	***	ns	*	ns
Psychedelic Experience Scale (PES48)																
Mystical	0 ± 0	6.6 ± 2.0	23 ± 5.4	35 ± 4.7	38 ± 5.9	< 0.001	ns	***	***	***	*	***	***	ns	*	ns
Positive mood	0.5 ± 0.5	19 ± 3.9	44 ± 6.3	49 ± 6.2	52 ± 7.3	< 0.001	*	***	***	***	**	***	***	ns	ns	ns
Transcendence of time/space	0 ± 0	13 ± 4.0	46 ± 6.9	70 ± 5.2	85 ± 3.2	< 0.001	ns	***	***	***	***	***	***	***	***	*
Ineffability	0 ± 0	18 ± 5.4	44 ± 7.9	65 ± 6.7	82 ± 6.2	< 0.001	*	***	***	***	***	***	***	**	***	ns
MEQ30 total score	0.1 ± 0.1	14 ± 3.2	39 ± 5.9	55 ± 4.5	64 ± 4.6	< 0.001	*	***	***	***	***	***	***	**	***	ns
Mystical Experience	0 ± 0	0 ± 0	15 ± 8.2	5.0 ± 5.0	15 ± 8.2	ns										
Paradoxicality	0 ± 0	6.0 ± 2.7	26 ± 4.9	42 ± 4.0	56 ± 4.5	< 0.001	ns	***	***	***	***	***	***	**	***	*
Connectedness	0 ± 0	13 ± 4.0	32 ± 5.0	39 ± 5.6	41 ± 6.9	< 0.001	ns	***	***	***	**	***	***	ns	ns	ns
Visual experience	0 ± 0	12 ± 2.5	40 ± 5.	54 ± 5.1	62 ± 5.7	< 0.001	ns	***	***	***	***	***	***	ns	***	ns
Distressing experience	0.2 ± 0.2	8.2 ± 2.6	13 ± 4	23 ± 6.8	30 ± 7.3	< 0.001	ns	ns	***	***	ns	*	**	ns	*	ns
Near Death Experience Scale (NDE-C)																
Harmony	0 ± 0	8.4 ± 1.7	21 ± 3.4	23 ± 3.6	25 ± 4.1	< 0.001	ns	***	***	***	**	**	***	ns	ns	ns
Beyond the usual	0 ± 0	2.0 ± 0.5	6.5 ± 1.0	8.9 ± 0.8	11 ± 0.6	< 0.001	ns	***	***	***	***	***	***	*	***	**
Insight	0 ± 0	0.4 ± 0.3	2.0 ± 0.6	4.2 ± 0.9	4.8 ± 1.0	< 0.001	ns	ns	***	***	ns	***	***	ns	*	ns
Border	0 ± 0	0.9 ± 0.4	3.6 ± 0.9	5.9 ± 1.1	7.6 ± 1.1	< 0.001	ns	**	***	***	ns	***	***	ns	***	ns
Gateway	0 ± 0	1.2 ± 0.7	7.2 ± 2.7	10 ± 2.1	13 ± 3.5	< 0.001	ns	*	**	***	ns	**	***	ns	ns	ns
NDE-C total score	0 ± 0	7.4 ± 1.6	24 ± 3.9	36 ± 3.8	44 ± 3.4	< 0.001	ns	***	***	***	***	***	***	*	***	ns

*P<0.05, **P<0.01, ***P<0.001; ns, not significant; SEM, standard error of mean.

Table S4. Comparison of acute subjective effects and autonomic effects between the double-blinded randomized and the dose-escalated study arm for each dose level

		Placebo			5 mg			10 mg			15 mg			20 mg			25 mg
Study arm		R	E		R	E		R	E		R	E		R	E		E
Number of participants		20	16		20	16		20	16		20	15		20	12		10
		mean ± SEM (% score)	mean ± SEM (% score)	*p*	mean ± SEM (% score)	mean ± SEM (% score)	*p*	mean ± SEM (% score)	mean ± SEM (% score)	*p*	mean ± SEM (% score)	mean ± SEM (% score)	*p*	mean ± SEM (% score)	mean ± SEM (% score)	*p*	mean ± SEM (% score)
Subjective effects																	
Any drug effect	ΔE_{max}	0.0 ± 0.0	0.2 ± 0.2	ns	4.5 ± 0.5	4.2 ± 0.4	ns	8.2 ± 0.5	7.3 ± 0.4	ns	9.5 ± 0.2	9.0 ± 0.3	ns	9.9 ± 0.1	9.7 ± 0.2	ns	9.8 ± 0.1
	AUEC	0.0 ± 0.0	0.8 ± 0.8	ns	21 ± 3.5	25 ± 5.3	ns	51 ± 5.7	45 ± 4.5	ns	80 ± 8.8	74 ± 11	ns	103 ± 10	84 ± 11	ns	115 ± 15
Good drug effect	ΔE_{max}	0.0 ± 0.0	0.0 ± 0.0	ns	4.1 ± 0.4	3.6 ± 0.6	ns	6.5 ± 0.71	6.8 ± 0.43	ns	7.3 ± 0.7	7.2 ± 0.86	ns	7.9 ± 0.7	8.3 ± 0.7	ns	9.5 ± 0.3
	AUEC	0.0 ± 0.0	0.0 ± 0.0	ns	20 ± 3.5	24 ± 6.7	ns	42 ± 6.6	42 ± 4.9	ns	63 ± 9.9	62 ± 13	ns	80 ± 12	80 ± 13	ns	124 ± 25
Bad drug effect	ΔE_{max}	0.0 ± 0.0	0.4 ± 0.4	ns	1.1 ± 0.4	1.6 ± 0.5	ns	1.6 ± 0.5	1.8 ± 0.7	ns	2.4 ± 0.7	1.2 ± 0.4	ns	2.7 ± 0.9	1.4 ± 0.6	ns	2.5 ± 1.2
	AUEC	0.0 ± 0.0	1.5 ± 1.5	ns	4.3 ± 1.9	4.0 ± 1.3	ns	6.3 ± 2.5	4.2 ± 1.7	ns	13 ± 4.2	5.1 ± 1.9	ns	20 ± 6.4	5.6 ±2.6	ns	11 ± 5.8
Fear	ΔE_{max}	0.2 ± 0.1	0.5 ± 0.3	ns	1.0 ± 0.4	0.9 ± 0.5	ns	1.3 ± 0.4	0.8 ± 0.3	ns	2.0 ± 0.7	1.0 ± 0.5	ns	1.9 ± 0.8	0.8 ± 0.3	ns	1.4 ± 1.0
	AUEC	0.9 ± 0.8	12 ± 10	ns	3.3 ± 1.3	1.8 ± 1.0	ns	5.3 ± 2.6	1.8 ± 0.9	ns	10 ± 3.8	4.7 ± 2.8	ns	13 ± 5.6	2.8 ± 1.6	ns	5.6 ± 4.7
Autonomic effects																	
Systolic blood pressure (mmHg)	E_{max}	124 ± 3.0	127 ± 2.3	ns	130 ± 2.8	136 ± 3.1	ns	138 ± 3.7	141 ± 3.3	ns	140 ± 3.9	146 ± 3.3	ns	145 ± 3.5	153 ± 4.2	ns	148 ± 3.4
Diastolic blood pressure (mmHg)	E_{max}	77 ± 1.2	80 ± 1.7	ns	82 ± 1.6	86 ± 2.1	ns	87 ± 1.9	89 ± 2.3	ns	89 ± 2.4	90 ± 2.7	ns	92 ± 2.3	94 ± 4.3	ns	93 ± 2.3
Heart rate (beats/min)	E_{max}	84 ± 2.7	82 ± 2.7	ns	91 ± 3.4	92 ± 3.4	ns	92 ± 3.3	98 ± 4.4	ns	102 ± 4.5	102 ± 4.5	ns	108 ± 3.7	110 ± 5.3	ns	112 ± 6.5
RPP (mmHg x bpm)	E_{max}	10121 ± 368	10297 ± 448	ns	11609 ± 533	12427 ± 516	ns	12346 ± 447	13730 ± 638	ns	14066 ± 743	14447 ± 641	ns	15678 ± 744	16786 ± 825	ns	16205 ± 1209

*p<0.05, **p<0.01, ***p<0.001; ns, not significant; AUEC, Area under the effect-time curve; ΔEmax, maximal effect difference from baseline; E, Escalated study arm; RPP, Rate pressure product; R, Randomized study arm

Table S5. Comparison between the randomized and dose-escalation study arm for the the acute subjective effects on the 3 Dimensions of Altered States of Consciousness (3D-ASC) Scale, the Psychedelic Experience Scale (PES48), and Near Death Experience Scale (NDE-C)

	Placebo			5 mg			10 mg			15 mg			20 mg			25 mg
Study arm	R	E		R	E		R	E		R	E		R	E		E
Number of participants	20	16		20	16		20	16		20	15		20	12		10
	mean ± SEM (% score)	mean ± SEM (% score)	*p*	mean ± SEM (% score)	mean ± SEM (% score)	*p*	mean ± SEM (% score)	mean ± SEM (% score)	*p*	mean ± SEM (% score)	mean ± SEM (% score)	*p*	mean ± SEM (% score)	mean ± SEM (% score)	*p*	mean ± SEM (% score)
Altered States of Consciousness (3D-ASC) Scale																
OAV total Score	0.0 ± 0.0	0.2 ± 0.2	**	6.5 ± 1.6	7.0 ± 2.9	ns	31 ± 4.2	18 ± 3.5	*	45 ± 3.5	29 ± 4.5	**	51 ± 3.2	33 ± 5.0	**	46 ± 4.9
Oceanic boundlessness	0.0 ± 0.0	0.1 ± 0.1	ns	6.2 ± 2.0	7.0 ± 4.2	ns	31 ± 6.1	16 ± 5.0	*	42 ± 5.0	28 ± 6.2	*	48 ± 5.6	43 ± 7.5	ns	65 ± 7.8
Anxious ego-dissolution	0.0 ± 0.0	0.6 ± 0.5	ns	5.2 ± 1.6	5.1 ± 1.9	ns	22 ± 3.9	9.5 ± 2.9	**	37 ± 5.4	19 ± 5.5	*	47 ± 5.8	14 ± 2.9	***	14 ± 3.3
Visionary restructuralization	0.0 ± 0.0	0.0 ± 0.0	ns	8.3 ± 2.0	9.0 ± 3.4	ns	40 ± 4.4	28 ± 4.9	ns	57 ± 4.2	39 ± 5.5	*	59 ± 4.6	41 ± 6.2	*	54 ± 4.4
Experience of unity	0.0 ± 0.0	0.0 ± 0.0	ns	4.1 ± 1.8	7.0 ± 5.0	ns	30 ± 6.7	15 ± 5.7	*	48 ± 6.5	28 ± 7.2	*	54 ± 7.4	44 ± 9.5	ns	62 ± 9.2
Spiritual experience	0.0 ± 0.0	0.0 ± 0.0	ns	4.4 ± 1.4	6.6 ± 3.6	ns	22 ± 5.6	13 ± 4.6	ns	29 ± 5.4	25 ± 5.8	ns	33 ± 5.2	38 ± 7.2	ns	70 ± 12
Blissful state	0.0 ± 0.0	0.3 ± 0.3	ns	14 ± 3.9	8.9 ± 4.1	ns	41 ± 7.6	20 ± 7.2	ns	44 ± 7.5	28 ± 8.3	ns	41 ± 8.5	52 ± 10	ns	80 ± 7.2
Insightfulness	0.0 ± 0.0	0.0 ± 0.0	ns	5.0 ± 3.6	2.6 ± 1.6	ns	13 ± 3.7	6.8 ± 2.8	ns	19 ± 4.8	11 ± 3.7	ns	21 ± 5.2	30 ± 7.8	ns	49 ± 8.4
Disembodiment	0.0 ± 0.0	0.1 ± 0.1	ns	3.9 ± 1.7	7.6 ± 5.4	ns	41 ± 7.6	23 ± 5.8	ns	58 ± 6.3	43 ± 9.2	ns	74 ± 5.9	45 ± 10	*	64 ± 10
Impaired control and cognition	0.0 ± 0.0	1.0 ± 0.9	ns	6.8 ± 2.2	7.5 ± 2.7	ns	31 ± 5.0	13 ± 3.9	**	48 ± 5.5	22 ± 4.6	**	61 ± 5.4	22 ± 5.2	***	23 ± 5.6
Anxiety	0.0 ± 0.0	0.1 ± 0.1	ns	3.3 ± 1.3	2.4 ± 1.1	ns	12 ± 4.3	5.4 ± 2.2	ns	25 ± 7.3	16 ± 7.6	ns	31 ± 7.8	4.3 ± 1.4	ns	4.4 ± 2
Complex imagery	0.0 ± 0.0	0.0 ± 0.0	ns	6.4 ± 2.6	4.5 ± 3.0	ns	42 ± 8.4	27 ± 7.9	ns	64 ± 7.1	47 ± 7.9	ns	65 ± 6.7	52 ± 11	ns	76 ± 5.2
Elementary imagery	0.0 ± 0.0	0.0 ± 0.0	ns	19 ± 5.1	17 ± 5.9	ns	66 ± 6.1	49 ± 6.1	ns	80 ± 4.2	59 ± 7.6	ns	83 ± 5.5	58 ± 7	**	73 ± 5.5
Audio-visual synesthesia	0.0 ± 0.0	0.1 ± 0.1	ns	7.0 ± 2.8	14 ± 6.5	ns	42 ± 7.2	27 ± 8.6	ns	65 ± 6.8	44 ± 10	ns	67 ± 8.0	38 ± 8.4	*	50 ± 9.1
Changed meaning of percepts	0.0 ± 0.0	0.0 ± 0.0	ns	1.8 ± 0.7	3.0 ± 1.4	ns	20 ± 5.2	14 ± 6.0	ns	32 ± 6.7	14 ± 3.4	ns	37 ± 6.1	20 ± 4.3	ns	31 ± 7.3
Psychedelic Experience Scale (PES48)																
Mystical	0.0 ± 0.0	0.0 ± 0.0	ns	6.6 ± 2.0	10 ± 5.3	ns	23 ± 5.4	23 ± 5.9	ns	35 ± 4.7	32 ± 7.3	ns	38 ± 5.9	42 ± 7.7	ns	67 ± 8.6
Positive mood	0.5 ± 0.5	0.4 ± 0.3	ns	19 ± 3.9	18 ± 5.6	ns	44 ± 6.3	36 ± 6.2	ns	49 ± 6.2	39 ± 7.4	ns	52 ± 7.3	55 ± 7.1	ns	76 ± 6.9
Transcendence of time/space	0.0 ± 0.0	1.0 ± 1.0	ns	13 ± 4.0	18 ± 6.4	ns	46 ± 6.9	48 ± 7.3	ns	70 ± 5.2	60 ± 7.7	ns	85 ± 3.2	63 ± 8.8	*	77 ± 5.8
Ineffability	0.0 ± 0.0	0.0 ± 0.0	ns	18 ± 5.4	9.6 ± 2.4	ns	44 ± 7.9	34 ± 5.9	ns	65 ± 6.7	63 ± 7.1	ns	82 ± 6.2	64 ± 8.8	*	77 ± 6.8
MEQ30 total score	0.1 ± 0.1	0.4 ± 0.3	ns	14 ± 3.2	14 ± 4.4	ns	39 ± 5.9	35 ± 4.1	ns	55 ± 4.5	49 ± 6.2	ns	64 ± 4.6	56 ± 7.1	ns	74 ± 5.7
Mystical Experience	0 ± 0	0 ± 0	ns	0 ± 0	0 ± 0	ns	15 ± 8.2	0 ± 0	ns	5.0 ± 5.0	7.0 ± 7.0	ns	15 ± 8.2	17 ± 11	ns	60 ± 16
Paradoxicality	0.0 ± 0.0	0.0 ± 0.0	ns	6.0 ± 2.7	6.8 ± 3.9	ns	26 ± 4.9	24 ± 4.9	ns	42 ± 4.0	37 ± 6.6	ns	56 ± 4.5	39 ± 8.4	ns	47 ± 7.3
Connectedness	0.0 ± 0.0	0.0 ± 0.0	ns	13 ± 4.0	12 ± 4.4	ns	32 ± 5.0	25 ± 5.8	ns	39 ± 5.6	32 ± 7.5	ns	41 ± 6.9	43 ± 7.7	ns	67 ± 6.1
Visual experience	0.0 ± 0.0	0.0 ± 0.0	ns	12 ± 2.5	20 ± 4.4	ns	40 ± 5.1	47 ± 5.6	ns	54 ± 5.1	48 ± 5.6	ns	62 ± 5.7	52 ± 6.4	ns	56 ± 7.3
Distressing experience	0.2 ± 0.2	1.5 ± 1.1	ns	8.2 ± 2.6	5.8 ± 1.7	ns	13 ± 4.0	8.0 ± 3.1	ns	23 ± 6.8	22 ± 6.8	ns	30 ± 7.3	8 ± 2.8	ns	6.8 ± 4.6
Near Death Experience scale (NDE-C)																
Harmony	0.0 ± 0.0	0.8 ± 0.5	ns	8.4 ± 1.7	8.2 ± 3.0	ns	21 ± 3.4	18 ± 4.0	ns	23 ± 3.6	18 ± 4.1	ns	25 ± 4.1	30 ± 4.2	ns	38 ± 3.9
Beyond the usual	0.0 ± 0.0	0.0 ± 0.0	ns	2.0 ± 0.5	2.6 ± 0.8	ns	6.5 ± 1.0	6.1 ± 0.9	ns	8.9 ± 0.8	9.3 ± 1	ns	11 ± 0.6	8.2 ± 1.1	*	10 ± 1.1
Insight	0.0 ± 0.0	0.0 ± 0.0	ns	0.4 ± 0.3	0.9 ± 0.5	ns	2.0 ± 0.6	2.2 ± 0.8	ns	4.2 ± 0.9	4.3 ± 1	ns	4.8 ± 1.0	4.7 ± 1.1	ns	7.8 ± 1.1
Border	0.0 ± 0.0	0.1 ± 0.1	ns	0.9 ± 0.4	1.0 ± 0.4	ns	3.6 ± 0.9	2.9 ± 0.7	ns	5.9 ± 1.1	5.5 ± 1.2	ns	7.6 ± 1.1	5.1 ± 1	ns	6.5 ± 1.5
Gateway	0.0 ± 0.0	0.0 ± 0.0	ns	1.2 ± 0.7	3.5 ± 1.9	ns	7.2 ± 2.7	6.2 ± 2.6	ns	10 ± 2.1	9.2 ± 3	ns	13 ± 3.5	9.9 ± 3.5	ns	16 ± 4.8
NDE-C total score	0.0 ± 0.0	0.3 ± 0.2	ns	7.4 ± 1.6	9.3 ± 3.2	ns	24 ± 3.9	22 ± 3.2	ns	36 ± 3.8	35 ± 4.4	ns	44 ± 3.4	35 ± 4.9	ns	47 ± 5.7

*P<0.05, **P<0.01, ***P<0.001; ns, not significant; E, Escalation; R, Randomized, SEM, standard error of mean.

Table S6. Acute adverse drug effects

Complaints	Randomized (n=20)		Escalated (n=16)	
	0 min	270 min	0 min	330 min
Anxiety	0	8	0	4
Headache	2	9	0	3
Feeling of weekness	0	6	2	3
Palpitation	1	6	1	2
Impaired concentration	0	4	0	2
Fear of death	0	4	0	0
Dyspnoea	0	4	0	2
Tiredness	10	13	3	8
Feeling dull	1	4	0	4
Throat thightness	0	3	0	1
Hot flashes	0	3	0	0
Dry mouth	1	4	1	2
Tendency to cry	0	3	0	6
Lack of appetite	0	2	0	0
Feeling exhausted	0	2	0	3
Lack of energy	0	2	0	4
Perspiration	0	2	0	0
Heavy legs	0	2	1	1
Negative thoughts	0	2	0	1
Shivering	1	3	0	4
Numbness in hands and feet	0	2	1	3
Dysphagia	0	1	0	0
Chest pain	0	1	0	0
Hypersensitivity to certain odors	0	1	0	0
Nausea	0	1	0	2
Not feeling at ease	2	3	0	1
Urge to urinate	2	3	0	3
Hypersensitivity to cold	0	1	0	0
Increased need to sleep	0	1	0	0
Intermittent shortness of breath	0	1	0	2
Freezing	1	2	1	1
Increased appetite	0	1	0	3
Inner tension	5	6	5	3
Cough	3	4	0	0
Hiccups	0	0	0	0
Feeling of pressure or abdominal fullness	0	0	0	0
Abdominal pain	0	0	1	0
Fainting	0	0	0	0
Dyspepsia	0	0	0	0
Vomiting	0	0	0	0
Diarrhea	0	0	0	0
Back pain	0	0	2	0
Feeling restless	5	5	1	2
Impaired balance	0	0	1	0
Erotic thoughts	0	0	0	0
Hypersensitivity to warm	0	0	0	0
Bad dreams	0	0	0	0
Pruritus	0	0	0	0
Blushing	0	0	2	1
Dizziness	1	1	0	2
Feeling suffocated	0	0	0	0
Decrease in body weight	0	0	0	0
Increase in body weight	0	0	0	0
Forgetfulness	0	0	0	0
Bruxism	1	1	1	2
Constipation	1	0	0	0
Irritability	1	0	0	0
Joint and limb pain	2	1	1	0
Restless legs	1	0	0	2
Decreased libido	1	0	1	0
Sore/scratchy throat	2	1	0	1
Brooding	3	1	0	2
Insomnia	2	0	1	1
Job-related or personal problems	2	0	2	2
Neck pain	3	1	0	0
Cold feet	5	1	2	2
**Total count**	59	126	30	85

Data indicate number of subjects reporting an effect.

Table S7. Pharmacokinetic parameters for DMT based on non-compartmental analyses in the dose-escalation study arm arm [geometric mean (95%CI), range]

Condition	5 mg	10 mg	15 mg	20 mg	25 mg
Number of participants	15*	15*	14*	11*	9*
C_{max} (ng/ml)	8.2 (7.0–9.6)	20 (16–26)	35 (27–45)	51 (34–76)	53 (40–70)
	5.0–13	8.8–54	19–105	23–161	28–82
T_{max} (min)	2.4 (2.0–2.9)	2.3 (2.0–2.7)	2.1 (1.9–2.3)	2.1 (1.9–2.5)	2.2 (1.8–2.6)
	2–4	2–4	2–4	2–4	2–4
$t_{1/2}$ (min)	5.6 (4.9–6.5)	6.3 (5.4–7.3)	6.1 (5.1–7.3)	7 (5.7–8.7)	6.8 (5.7–8.2)
	3.6–8.3	4.1–9.3	3.6–13	4.8–13	5.1–9.7
CL (L/min)	57 (46–70)	45 (35–57)	42 (33–53)	38 (27–54)	43 (31–59)
	32–117	23–113	21–83	17–86	23–90
V_z (L)	460 (381–555)	404 (336–486)	370 (308–445)	383 (303–483)	420 (325–544)
	244–718	266–779	239–590	244–639	262–663
AUC_{∞} (ng x min/ml)	88 (71–109)	224 (174–288)	358 (283–451)	527 (372–745)	586 (423–810)
	43–157	89–430	181–708	234–1210	277–1109

AUC, area under the plasma concentration–time curve; AUC∞, AUC from time zero to infinity; CL apparent total clearance; Cmax, maximum observed plasma concentration; t1/2, plasma elimination half–life; Tmax, time to reach Cmax; 95%CI, 95% confidence interval; Vz, apparent volume of distribution.

*One participant was excluded from all analyses due to missing plasma samples and particpants could end at each level of the dose escalation.

Table S8. Mean values of acute subjective effects ordered by dose condition and dose number in the randomized study arm

Condition		5 mg						10 mg						15 mg						20 mg					
Dose number		1	2	3	4	5		1	2	3	4	5		1	2	3	4	5		1	2	3	4	5	
Participants (n)		5	3	4	4	4		4	5	5	5	2		3	6	4	4	3		4	3	5	2	6	
Any drug effect	ΔEmax*	5.2 ± 1.0	4.0 ± 1.5	4.0 ± 0.9	4.0 ± 0.7	4.8 ± 1.9	ns	7.2 ± 1.5	7.2 ± 1.2	9 ± 0.6	9.2 ± 0.8	8.0 ± 1.0	ns	8.7 ± 0.7	9.5 ± 0.5	9.8 ± 0.2	10 ± 0.0	9.0 ± 0.6	ns	9.5 ± 0.5	10 ± 0.0	10 ± 0.0	10 ± 0.0	10 ± 0.0	ns
	AUEC*	20 ± 3.8	31 ± 19	20 ± 6.7	19 ± 5.9	20 ± 8.9	ns	57 ± 24	46 ± 8.2	48 ± 3.3	50 ± 9.7	65 ± 35	ns	51 ± 3.5	83 ± 10.9	115 ± 34	81 ± 15	54 ± 3.0	ns	81 ± 8.7	124 ± 31	114 ± 21	161 ± 64	78 ± 6.9	ns
Good drug effect	ΔEmax*	5.4 ± 0.9	4.0 ± 1.0	4.0 ± 0.9	3.8 ± 0.6	3.0 ± 1.2	ns	7.8 ± 1.0	6.8 ± 1.2	6.4 ± 1.8	4.8 ± 2.3	7.0 ± 1.0	ns	5.7 ± 1.9	6.5 ± 1.4	8.5 ± 0.9	6.8 ± 2.0	9.3 ± 0.3	ns	9.5 ± 0.5	8.3 ± 1.7	6.6 ± 1.5	8.5 ± 0.5	7.3 ± 1.8	ns
	AUEC*	22 ± 5.1	27 ± 19	23 ± 9.1	17 ± 4.6	14.0 ± 5.6	ns	58 ± 23	42 ± 6.4	36 ± 9.7	23 ± 12	61 ± 36	ns	40 ± 15	50 ± 13	110 ± 32	57 ± 23	57 ± 2.8	ns	70 ± 8.7	107 ± 39	77 ± 24	143 ± 74	55 ± 17	ns
Bad drug effect	ΔEmax*	2.2 ± 0.8	0.7 ± 0.7	0.0 ± 0.0	0.5 ± 0.5	1.8 ± 1.8	ns	3.8 ± 1.3	0.8 ± 0.6	1.6 ± 1	0.5 ± 0.5	1.5 ± 0.5	ns	2.7 ± 1.5	3.0 ± 1.6	3.8 ± 2.2	1.5 ± 1.0	0.0 ± 0.0	ns	2.5 ± 2.5	7.7 ± 1.5	0.6 ± 0.6	1.0 ± 1.0	2.7 ± 1.7	ns
	AUEC*	5.0 ± 1.7	8.3 ± 8.3	0.0 ± 0.0	1.8 ± 1.8	7.2 ± 7.2	ns	19 ± 10	3.8 ± 2.9	4 ± 2.8	1.5 ± 1.5	3.0 ± 1.0	ns	11 ± 5.8	17 ± 8.9	26 ± 14	8.5 ± 5.7	0.0 ± 0.0	ns	11 ± 11	54 ± 14	2.4 ± 2.4	23 ± 23	22 ± 15	ns
Fear	ΔEmax*	1.6 ± 0.5	0.3 ± 0.3	0.0 ± 0.0	0.8 ± 0.8	1.8 ± 1.4	ns	2.2 ± 1.3	1.6 ± 1.1	0.4 ± 0.4	0.8 ± 0.8	1.0 ± 0.0	ns	2.0 ± 1.5	1.2 ± 0.6	3.8 ± 2.2	2.5 ± 2.5	0.3 ± 0.3	ns	2.5 ± 2.5	2.3 ± 2.3	0.6 ± 0.6	1.0 ± 1.0	2.5 ± 1.7	ns
	AUEC*	3.2 ± 1.0	7.7 ± 7.7	0.0 ± 0.0	3.0 ± 3.0	3.5 ± 2.9	ns	16 ± 12	3.6 ± 2.2	1.6 ± 1.6	3.2 ± 3.2	2.0 ± 0.0	ns	9.0 ± 7.5	6.7 ± 4.5	25 ± 13	10 ± 10	0.7 ± 0.7	ns	11 ± 11	14 ± 14	2.4 ± 2.4	23 ± 23	20 ± 15	ns

AUEC, Area under the effect-time curve; ΔEmax, maximal effect difference from baseline; n, number; ns, not significant; SEM, standard error of mean.

* All Emax and AUEC values are expressed as mean ± SEM. P-values were calculated using one-way ANOVA with dose number as a factor.

Table S9. Drug dose identification after each administration and at the end of the study in the double-blinded, randomized treatment study arm

	Placebo		5 mg		10 mg		15 mg		20 mg	
	After administration	After study	After administration	After study	After administration	After study	After administration	After study	After administration	After study
correctly identified, n (%)	100.0%	80.0%	87.5%	85.0%	43.8%	70.0%	68.8%	70.0%	56.2%	85.0%
missclassified as placebo, n (%)			6.2%	5.0%	0.0%	5.0%	0.0%	5.0%	0.0%	5.0%
missclassified as 5 mg, n (%)	0.0%	0.0%			25.0%	10.0%	0.0%	5.0%	0.0%	0.0%
missclassified as 10 mg, n (%)	0.0%	10.0%	6.2%	5.0%			18.8%	15.0%	0.0%	0.0%
missclassified as 15 mg, n (%)	0.0%	5.0%	0.0%	5.0%	12.5%	10.0%			43.8%	10.0%
missclassified as 20 mg, n (%)	0.0%	5.0%	0.0%	0.0%	18.8%	5.0%	12.5%	5.0%		

After administration = 30 minutes after bolus administration; After study = at the end of study visit; *n* = 20.

References

1 Riba J, McIlhenny EH, Bouso JC, Barker SA. Metabolism and urinary disposition of N,N-dimethyltryptamine after oral and smoked administration: a comparative study. Drug Test Anal. 2015;7:401-6.

2 Vogt SB, Ley L, Erne L, Straumann I, Becker AM, Klaiber A, et al. Acute effects of intravenous DMT in a randomized placebo-controlled study in healthy participants. Transl Psychiatry. 2023;13:172.

3 Erne L, Vogt SB, Muller L, Nuraj A, Becker A, Klaiber A, et al. Acute dose-dependent effects and self-guided titration of continuous N,N-dimethyltryptamine infusions in a double-blind placebo-controlled study in healthy participants. Neuropsychopharmacology. 2025;50:1008-16.

4 Holze F, Duthaler U, Vizeli P, Muller F, Borgwardt S, Liechti ME. Pharmacokinetics and subjective effects of a novel oral LSD formulation in healthy subjects. Br J Clin Pharmacol. 2019;85:1474-83.

5 Dittrich A. The standardized psychometric assessment of altered states of consciousness (ASCs) in humans. Pharmacopsychiatry. 1998;31 (Suppl 2):80-4.

6 Studerus E, Gamma A, Vollenweider FX. Psychometric evaluation of the altered states of consciousness rating scale (OAV). PLoS One. 2010;5:e12412.

7 Liechti ME, Dolder PC, Schmid Y. Alterations in conciousness and mystical-type experiences after acute LSD in humans. Psychopharmacology. 2017;234:1499-510.

8 Carhart-Harris RL, Kaelen M, Bolstridge M, Williams TM, Williams LT, Underwood R, et al. The paradoxical psychological effects of lysergic acid diethylamide (LSD). Psychol Med. 2016;46:1379-90.

9 Schmid Y, Enzler F, Gasser P, Grouzmann E, Preller KH, Vollenweider FX, et al. Acute effects of lysergic acid diethylamide in healthy subjects. Biol Psychiatry. 2015;78:544-53.

10 Dolder PC, Schmid Y, Mueller F, Borgwardt S, Liechti ME. LSD acutely impairs fear recognition and enhances emotional empathy and sociality. Neuropsychopharmacology. 2016;41:2638-46.

11 Holze F, Vizeli P, Muller F, Ley L, Duerig R, Varghese N, et al. Distinct acute effects of LSD, MDMA, and D-amphetamine in healthy subjects. Neuropsychopharmacology. 2020;45:462-71.

12 Bershad AK, Schepers ST, Bremmer MP, Lee R, de Wit H. Acute subjective and behavioral effects of microdoses of lysergic acid diethylamide in healthy human volunteers. Biol Psychiatry. 2019;86:792-800.

13 Preller KH, Herdener M, Pokorny T, Planzer A, Kraehenmann R, Stämpfli P, et al. The fabric of meaning and subjective effects in LSD-induced states depend on serotonin 2A receptor activation Curr Biol. 2017;27:451-57.

14 Roseman L, Nutt DJ, Carhart-Harris RL. Quality of acute psychedelic experience predicts therapeutic efficacy of psilocybin for treatment-resistant depression. Front Pharmacol. 2017;8:974.

15 Griffiths RR, Johnson MW, Carducci MA, Umbricht A, Richards WA, Richards BD, et al. Psilocybin produces substantial and sustained decreases in depression and anxiety in patients with life-threatening cancer: a randomized double-blind trial. J Psychopharmacol. 2016;30:1181-97.

16 Stocker K, Hartmann M, Ley L, Becker AM, Holze F, Liechti ME. The revival of the psychedelic experience scale: revealing its extended-mystical, visual, and distressing experiential spectrum with LSD and psilocybin studies. J Psychopharmacol. 2024;38:80-100.

17 Griffiths RR, Richards WA, McCann U, Jesse R. Psilocybin can occasion mystical-type experiences having substantial and sustained personal meaning and spiritual significance. Psychopharmacology. 2006;187:268-83; discussion 84-92.

18 Barrett FS, Johnson MW, Griffiths RR. Validation of the revised Mystical Experience Questionnaire in experimental sessions with psilocybin. J Psychopharmacol. 2015;29:1182-90.

19 Becker AM, Klaiber A, Holze F, Istampoulouoglou I, Duthaler U, Varghese N, et al. Ketanserin reverses the acute response to LSD in a randomized, double-blind, placebo-controlled, crossover study in healthy participants. Int J Neuropsychopharmacol. 2023;26:97-106.

20 Holze F, Vizeli P, Ley L, Muller F, Dolder P, Stocker M, et al. Acute dose-dependent effects of lysergic acid diethylamide in a double-blind placebo-controlled study in healthy subjects. Neuropsychopharmacology. 2021;46:537-44.

21 Holze F, Ley L, Muller F, Becker AM, Straumann I, Vizeli P, et al. Direct comparison of the acute effects of lysergic acid diethylamide and psilocybin in a double-blind placebo-controlled study in healthy subjects. Neuropsychopharmacology. 2022;47:1180-87.

22 Straumann I, Ley L, Holze F, Becker AM, Klaiber A, Wey K, et al. Acute effects of MDMA and LSD co-administration in a double-blind placebo-controlled study in healthy participants. Neuropsychopharmacology. 2023;48:1840-48.

23 Martial C, Simon J, Puttaert N, Gosseries O, Charland-Verville V, Nyssen A-S, et al. The Near-Death Experience Content (NDE-C) scale: Development and psychometric validation. Consciousness and Cognition. 2020;86:103049.
